# Epidemiology, resistance genomics and susceptibility of *Acinetobacter* species: results from the 2020 Spanish nationwide surveillance study

**DOI:** 10.2807/1560-7917.ES.2024.29.15.2300352

**Published:** 2024-04-11

**Authors:** Cristina Lasarte-Monterrubio, Paula Guijarro-Sánchez, Isaac Alonso-Garcia, Michelle Outeda, Romina Maceiras, Lucia González-Pinto, Marta Martínez-Guitián, Carlos Fernández-Lozano, Juan Carlos Vázquez-Ucha, German Bou, Jorge Arca-Suárez, Alejandro Beceiro

**Affiliations:** 1 Microbiology Department, A Coruña University Hospital (CHUAC), Institute of Biomedical Research of A Coruña (INIBIC), Spain; 2 NANOBIOFAR, Centre for Research in Molecular Medicine and Chronic Diseases (CiMUS), Universidad de Santiago de Compostela, Santiago de Compostela, Spain; 3 Department of Computer Science and Information Technologies, Faculty of Computer Science, Research Center of Information and Communication Technologies (CITIC), University of A Coruña, A Coruña, Spain; 4 CIBER de Enfermedades Infecciosas (CIBERINFEC), A Coruña, Spain

**Keywords:** *Acinetobacter* spp., *Acinetobacter baumannii*, epidemiology, antimicrobial susceptibility, WGS, CHDLs

## Abstract

**Background:**

As increasing antibiotic resistance in *Acinetobacter baumannii* poses a global healthcare challenge, understanding its evolution is crucial for effective control strategies.

**Aim:**

We aimed to evaluate the epidemiology, antimicrobial susceptibility and main resistance mechanisms of *Acinetobacter* spp. in Spain in 2020, and to explore temporal trends of *A. baumannii*.

**Methods:**

We collected 199 single-patient *Acinetobacter* spp. clinical isolates in 2020 from 18 Spanish tertiary hospitals. Minimum inhibitory concentrations (MICs) for nine antimicrobials were determined. Short-read sequencing was performed for all isolates, and targeted long-read sequencing for *A. baumannii*. Resistance mechanisms, phylogenetics and clonality were assessed. Findings on resistance rates and infection types were compared with data from 2000 and 2010.

**Results:**

Cefiderocol and colistin exhibited the highest activity against *A. baumannii*, although colistin susceptibility has significantly declined over 2 decades. *A.* non-*baumannii* strains were highly susceptible to most tested antibiotics. Of the *A. baumannii* isolates, 47.5% (56/118) were multidrug-resistant (MDR). Phylogeny and clonal relationship analysis of *A. baumannii* revealed five prevalent international clones, notably IC2 (ST2, n = 52; ST745, n = 4) and IC1 (ST1, n = 14), and some episodes of clonal dissemination. Genes *bla*_OXA-23_, *bla*_OXA-58_ and *bla*_OXA-24/40_ were identified in 49 (41.5%), eight (6.8%) and one (0.8%) *A. baumannii* isolates, respectively. IS*Aba*1 was found upstream of the gene (a *bla*_OXA-51_-like_)_ in 10 isolates.

**Conclusions:**

The emergence of OXA-23-producing ST1 and ST2, the predominant MDR lineages, shows a pivotal shift in carbapenem-resistant *A. baumannii* (CRAB) epidemiology in Spain. Coupled with increased colistin resistance, these changes underscore notable alterations in regional antimicrobial resistance dynamics.

**Key public health message**

**What did you want to address in this study and why?**

The bacteria *Acinetobacter* species, especially *Acinetobacter baumannii*, pose a major therapeutic threat because of their ability to readily develop antimicrobial resistance. Our study, conducted in Spain in 2020, aimed to evaluate the spread of *Acinetobacter* primarily in hospital environments, its response to antibiotics, and the factors contributing to its resistance to treatment. We compared these findings with data from 2000 and 2010.

**What have we learnt from this study?**

Nearly 80% of the collected *Acinetobacter* spp. strains were related to infections, with *A. baumannii* being the most prevalent species. We observed that resistance of *A. baumannii* to most antibiotics either decreased or remained stable over time, except for colistin, a last resort agent. The enzyme OXA-23 has become the driver of beta-lactam (carbapenem) resistance in Spain, mainly linked to few *A. baumannii* lineages, which vary regionally.

**What are the implications of your findings for public health?**

High antibiotic resistance rates of *A. baumannii*, emerging resistance mechanisms, and the spread of closely related strains underscore the importance of active surveillance, robust control measures, accurate identification and swift diagnosis to safeguard therapeutic options against *Acinetobacter*.

## Introduction

The *Acinetobacter* genus encompasses diverse Gram-negative bacterial species, from environmental to pathogenic microbes. Representatives of the *Acinetobacter* *calcoaceticus*-*baumannii* (ACB) complex, particularly *A. baumannii*, are the most clinically important. Infections typically occur in patients with serious underlying conditions and often result from anatomical barrier breaches, leading to outcomes like aspiration pneumonia, soft tissue damage and catheter-related bacteraemia [1]. Antibiotic treatment spans from carbapenems (for susceptible *Acinetobacter*) to polymyxins, aminoglycosides, ampicillin/sulbactam and cefiderocol for carbapenem-resistant (CRAB) or extensively drug-resistant (XDR) *A. baumannii* isolates [1,2].

*Acinetobacter baumannii* possesses an exceptional ability to develop antimicrobial resistance via both horizontal gene acquisition and alteration of chromosomal mechanisms. Carbapenem non-susceptibility has been mainly related to the expression of acquired carbapenem-hydrolysing class D beta-lactamases (CHDLs), such as *bla*_OXA-23-like_, alongside reduced cell permeability and increased efflux system activity [3,4]. Resistance mechanisms have also been described concerning other antimicrobial agents used against XDR and CRAB isolates. Thus, sulbactam resistance can result from PBP3 mutations [5], *adeABC* efflux system overexpression [6] and the presence of OXA-23 and TEM-1 beta-lactamases [7]. Mutations in PmrAB, IS*Aba*1-mediated overexpression of the *pmrC* homologue *eptA* [8], and inactivation of lipid A biosynthesis-associated *lpx* genes [9], alter polymyxin (e.g. colistin) activity through modification or loss of the lipopolysaccharide (LPS). Aminoglycoside resistance is facilitated by aminoglycoside modifying enzymes (AMEs) and changes to the 16S rRNA structure. Additionally, the effectiveness of cefiderocol, a last-resort agent, may be compromised by downregulation or absence of siderophore receptors like PirA and PiuA [10], and potentially by the emergence of specific beta-lactamases such as PER and NDM [11].

Evaluation and management of trends in antimicrobial resistance are important aspects of all national or international surveillance studies and are relevant for guiding decisions towards appropriate treatment choices. Therefore, we aimed to analyse a collection of *Acinetobacter* spp., obtained from hospitals across Spain in 2020, to assess their resistance profiles and track epidemiological changes over time. Furthermore, our goal was to determine the national prevalence of resistance mechanisms to carbapenems, as well as to evaluate the occurrence of those associated with other therapeutic alternatives. This study provides continuity by juxtaposing current isolates with those gathered in Spain in two prior multicentre investigations conducted in 2000 (GEIH/REIPI-Ab-2000) [12,13] and 2010 (GEIH/REIPI-Ab-2010) [14,15], following similar inclusion criteria. These previous investigations unveiled the national epidemiology of *A. baumannii*, highlighting the increasing dominance of ST2, and revealed troubling trends in antimicrobial resistance such as the declining effectiveness of carbapenems and the crucial role of polymyxins. Here, we aimed to modernise and refine, via utilisation of whole genome sequencing (WGS), our understanding of *Acinetobacter* spp. epidemiology and its resistance mechanisms, encompassing also the frequently overlooked *A.* non-*baumannii* isolates.

## Methods

### Study design and setting

*Acinetobacter* spp. single-patient clinical isolates were prospectively recovered during a 4-month period (December 2019–March 2020). Sample collection occurred through a call to tertiary hospitals (n = 24), issued to ensure regional diversity and engage hospitals known for their pivotal role in specialised care. 

With collection of each isolate, a standard form (based on the previous studies [12-15]) was completed, requesting information on origin of isolates (geographical region and hospital were collected), date of isolation, patient age/sex, source/sample site of isolation, colonisation/infection status, type of infection if applicable, species-level identification method, and susceptibility profile or resistance mechanism, if available. No data on underlying conditions were collected. Ultimately, 18 of 24 facilities responded (see Supplementary Table S1 for a list of participating hospitals and number of isolates per site). The microbiology department of the A Coruña University Hospital (A Coruña, Spain) acted as the reference laboratory.

The results obtained in this multicentre study have been compared with the data on resistance rates and epidemiology of two previous studies, conducted in 2000 (GEIH/REIPI-Ab-2000 [12,13]) and 2010 (GEIH/REIPI-Ab-2010 [14,15]). 

### Bacterial isolates

*Acinetobacter* spp. clinical isolates were obtained from sampling of infected patients or as part of colonisation studies. For each bacterial strain, data such as geographic origin, type of infection, sex and age of the patient were recorded. Colonisation was defined as the presence of microorganisms without associated disease, either (i) in epidemiological surveillance samples or (ii) when, despite the presence of infection, another pathogen was identified in the sample as the causative agent of the infection according to clinical criteria. Infection was characterised by the isolation of a clinically relevant *Acinetobacter* strain in the sample. The isolates were frozen at −80 °C in Luria Bertani (LB) broth supplemented with 15% glycerol until analysis.

### Susceptibility testing

Minimum inhibitory concentrations (MIC) were determined by reference broth microdilution for antimicrobials imipenem, meropenem, sulbactam (as the representative of the ampicillin/sulbactam combination), cefepime, cefiderocol, amikacin, tobramycin, ciprofloxacin and colistin following Clinical and Laboratory Standards Institute (CLSI) guidelines [16]. 

The CLSI M100 ED32 breakpoints were consulted for interpretive purposes. These were chosen to enable comparison across three timepoints and because of the lack of provided susceptibility breakpoints for some antimicrobials (ampicillin/sulbactam, cefiderocol, cefepime) by the European Committee on Antimicrobial Susceptibility Testing (EUCAST) v12.0 (2022). The rates presented for 2000 and 2010 were adjusted to the current standards (M100 ED32) used in 2020.

Cefiderocol was provided by Shionogi (Osaka, Japan), and the other antibiotics were acquired from Sigma-Aldrich (Saint Louis, United States (US)). *Escherichia coli* ATCC 25922 and *Pseudomonas aeruginosa* ATCC 27853 were used as control strains. Sulbactam susceptibility breakpoints were extrapolated from those of ampicillin/sulbactam as follows: susceptible (S) ≤ 4 mg/L, intermediate (I) = 8 mg/L, and resistant (R) ≥ 16 mg/L.

### Whole genome sequencing and phylogenetic analysis

Total genomic DNA was extracted using the Wizard Genomic DNA Purification Kit (Promega). All *Acinetobacter* spp. isolates were fully sequenced using paired-end short reads on the Illumina MiSeq platform. Adapter sequences were removed using Trimmomatic v0.39 [17]. Moreover, a subset of 43 representatives of each *A. baumannii* clonal group were subjected to long-read sequencing with the MinION platform from Oxford Nanopore Technologies. Base-calling was performed using Guppy v6.5.7 (https://nanoporetech.com), and adapter trimming was conducted using Porechop v0.2.4 (https://github.com/rrwick/Porechop). Long-read sequencing was conducted with the purpose of evaluating the presence of insertion sequences (IS) involved in the overexpression of beta-lactamases, a frequent event in *A. baumannii* [18].

After quality control checks with FastQC v0.11.9 (http://www.bioinformatics.babraham.ac.uk/projects/fastqc), reads were de novo assembled with Unicycler v0.4.8. CheckM v1.1.6 [19] was used to assess the quality of the assembled genomes, which were annotated with Bakta v1.6 [20].

Species identification was carried out by in silico ribosomal multilocus sequence typing (rMLST), as described in Jolley et al. 2012 [21]. 

Genomic relatedness among the *A. baumannii* isolates was assessed using a distance matrix based on core genome multilocus sequence typing (cgMLST) allele calls. Isolates with ≤ 3 cgMLST distance were considered closely related. The neighbor-joining model was employed to construct the relative distances of the dendrogram. For *A.* non-*baumannii* species (*A. pittii* and *A. ursingii*), the putative core genome of the isolates was determined through pangenome clustering because of the unavailability of a public cgMLST scheme. The phylogenetic dendrogram was constructed following the GTR+G model (discrete GAMMA model of rate heterogeneity with four categories)*.* The reference genome selected for analysis of each *Acinetobacter* spp. were obtained from the ATCC Genome Portal (https://genomes.atcc.org) or retrieved from the NCBI reference genome of each species (https://www.ncbi.nlm.nih.gov/datasets/taxonomy)*. *Isolates that did not achieve 100% species identification through in silico rMLST were compared to the closest identified species. Additional details regarding the methodology employed can be found in the Supplementary Materials – Materials and Methods.

### Statistical analysis

R Commander v4.2.3 [22] was used to examine the disparities in colonisation, infection and infection types between *A. baumannii* and *A.* non-*baumannii* strains. Additionally, infection types and resistance rates of *A. baumannii* isolates from 2020 were compared to those from 2000 and 2010. The analysis employed the Pearson’s chi-squared test with a significance level set at p < 0.05.

## Results

A total of 199 *Acinetobacter* spp. isolates were recovered in 2020 from sources of infection (n = 159, 79.9%) or colonisation (n = 40, 20.1%). The region of origin of isolates and their distribution per hospital are displayed in Supplementary Table S1. The median age of the patient population was 60.6 years (range: 5 days–96 years), with a male-to-female ratio of 1.5:1. 

Remarkably, *A. baumannii* was the predominant species, accounting for 59.3% (n = 118) of all strains, while among the *A.* non-*baumannii* isolates *A. pittii* (14.1%, n = 28), *A. ursingii* (6.5%, n = 13) and *A. bereziniae* (4.5%, n = 9) were the more prevalent species (Table 1).

**Table 1:** *Acinetobacter* species included in the study, Spain, 2020 (n = 199)

*Acinetobacter* species	Number of isolates	Relative %
*Acinetobacter baumannii*	118	59.3
*Acinetobacter pittii*	28	14.1
*Acinetobacter ursingii*	13	6.5
*Acinetobacter bereziniae*	9	4.5
*Acinetobacter* spp^a^	6	3.0
*Acinetobacter calcoaceticus*	4	2.0
*Acinetobacter haemolyticus*	3	1.5
*Acinetobacter nosocomialis*	3	1.5
*Acinetobacter lactucae*	3	1.5
*Acinetobacter lwoffii*	2	1.0
*Acinetobacter junii*	2	1.0
*Acinetobacter guillouiae*	2	1.0
*Acinetobacter soli*	1	0.5
*Acinetobacter beijerinckii*	1	0.5
*Acinetobacter dispersus*	1	0.5
*Acinetobacter johnsonii*	1	0.5
*Acinetobacter radioresistens*	1	0.5
*Acinetobacter colistiniresistens*	1	0.5
**Total**	**199**	**100**

^a^ *Acinetobacter *spp. refers to isolates that did not reach 100% species identification by in silico ribosomal multilocus sequence typing (rMLST).

Among the analysed *Acinetobacter* spp. isolates, *A. baumannii* were significantly more associated with colonisation than the *A.* non-*baumannii* isolates (26.3%, n = 31 and 11.1%, n = 9, respectively; p = 0.0379).

**Figure 1:**
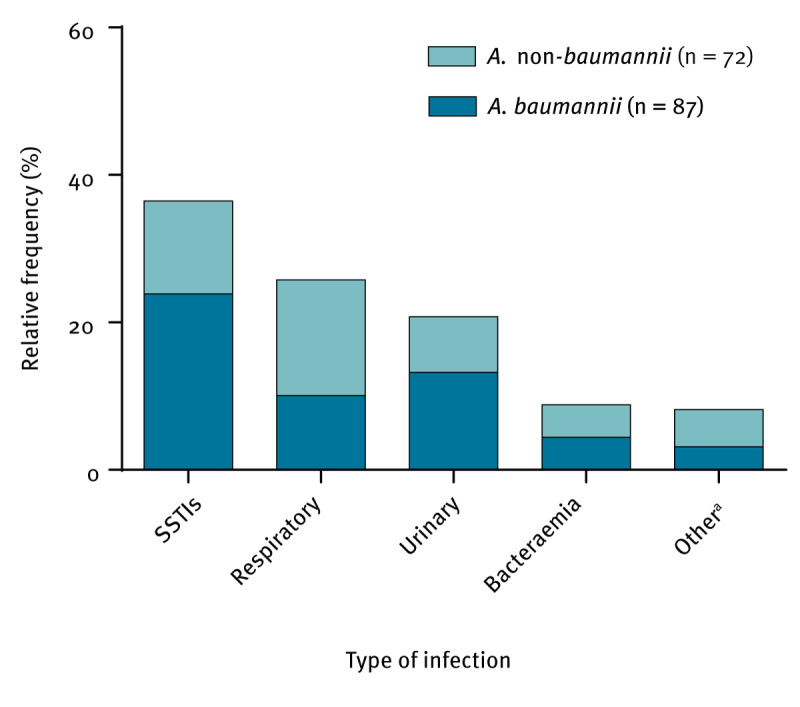
Distribution of the *Acinetobacter* species isolates by type of infection, Spain, 2020 (n = 159)

The most prevalent infection types for *Acinetobacter* spp. (n = 159) were skin and soft-tissue infections (SSTIs; 36.5%, n = 58), respiratory (25.8%, n = 41) and urinary (20.8%, n = 33) infections, followed by bacteraemia (8.8%, n = 14). Notably, *A.* non-*baumannii* isolates demonstrated a closer association with respiratory infections, whereas SSTIs emerged as the predominant infection type linked to *A. baumannii* (Figure 1). There was no specific correlation between the *Acinetobacter* species and the type of infection.

*A*.: *Acinetobacter*; SSTIs: skin and soft-tissue infections.

Number of isolates. *A*. non-*baumannii* (n = 72): SSTIs (n = 20), respiratory (n = 25), urinary (n = 12), bacteraemia (n = 7), other (n = 8). *A. baumannii *(n = 87): SSTIs (n = 38), respiratory (n = 16), urinary (n = 21), bacteraemia (n = 7), other (n = 5).

^a^ Other *Acinetobacter* infections were as follows: *A.* non-*baumannii*: intra-abdominal (n = 2), ear (n = 2), genital tract (n = 2), bone and joint (n = 1), not stated (n = 1); *A. baumannii*: disseminated (n = 1), intra-abdominal (n = 1), ear (n = 1), bone and joint (n = 1), prosthetic (n = 1).

Furthermore, upon retrospective analysis, there was a significant increase in the number of SSTIs caused by *A. baumannii* in 2020 (38/87 cases), notably higher than the occurrence in both 2000 (26/109 cases, p = 0.0372) and 2010 (31/151 cases, p = 0.0051) [13,15]. In contrast, the number of respiratory infections caused by *A. baumannii* (16/87 cases in 2020) decreased significantly (52/109, p = 0.0024 for 2000; and 73/151, p = 0.0013 for 2010). There has also been a surge in urinary infections (21/87 cases) since 2010 (15/151 cases, p = 0.0127).

### Susceptibility profiles among *Acinetobacter* species isolates

Cefiderocol (MIC_50/90_ =  ≤ 0.12/0.5 mg/L) and sulbactam (MIC_50/90_ = 0.5/8 mg/L) presented the highest activity against the *Acinetobacter* spp. collection, which showed resistance rates of 0% and 9.5%, respectively. By contrast, imipenem (MIC_50/90_ =  ≤ 0.25/32 mg/L) and meropenem (MIC_50/90_ = 0.5/ ≥ 64 mg/L) exhibited considerably lower activity, as ca 35% of the isolates were resistant to both antibiotics. The collection displayed a resistance rate of 11.6% to colistin, with MIC values spanning ≤ 0.12 to ≥ 128 mg/L (Table 2). However, these results were biased and highly dependent on the *Acinetobacter* species involved. While *A.* non-*baumannii* isolates generally demonstrated susceptibility to all antibiotics tested, *A. baumannii* isolates manifested resistance rates of around 60% for carbapenems and ciprofloxacin, 40–30% for aminoglycosides, and 14% for colistin (Table 3). Supplementary Tables S2 and S3 provide the MIC distribution and resistance rates for *A.* non-*baumannii* isolates and MIC values and genetic characteristics for each *A. baumannii* isolate, respectively.

**Table 2:** Minimum inhibitory concentrations and resistance rates of all *Acinetobacter* species isolates, Spain, 2020 (n = 199)

Class of antibiotic	Per cent of isolates at MIC (mg/L)												% R
	≤ 0.12	0.25	0.5	1	2	4	8	16	32	64	128	≥ 256	
Beta-lactams													
IMI	ND	59.3^a^	64.3	64.8	64.8	65.3	70.3	78.4	**98.0**	100**^b^**	ND	ND	34.6
MEM	ND	29.6^a^	56.8	64.8	64.8	64.8	67.3	71.4	79.4	**100^b^**	ND	ND	35.2
FEP	ND	ND	6.0^a^	25.1	51.3	60.8	64.3	70.3	85.9	**93.0**	100^b^	ND	29.6
FDC	70.3	86.4	**92.5**	97.5	100	ND	ND	ND	ND	ND	ND	ND	0
SUL^c^	ND	4.5^a^	51.3	62.3	68.8	74.4	**90.4**	97.5	99.5	100^b^	ND	ND	9.5
Aminoglycosides													
AMK	ND	ND	ND	15.1^a^	33.2	67.8	75.9	80.4	81.9	82.9	83.4	**100**	18.1
TOB	ND	ND	ND	68.3^a^	71.9	75.9	77.4	79.9	80.4	82.4	85.9	**100**	22.6
Fluoroquinolones													
CIP	ND	58.3 ^a^	61.3	62.8	63.8	63.8	64.8	65.3	70.8	**100^b^**	ND	ND	36.2
Polymyxins													
COL	3.0	9.0	33.2	64.8	87.9	**94.5**	98.5	99.0	99.5	99.5	100	ND	11.6

AMK: amikacin; CIP: ciprofloxacin; COL: colistin; FDC: cefiderocol; FEP: cefepime; IMI: imipenem; MEM: meropenem; MIC: minimum inhibitory concentration; ND: not determined; R: resistance; SUL: sulbactam; TOB: tobramycin.

^a^ Actual MIC value is equal or inferior to that stated.

^b^ Actual MIC value is equal or higher to that stated.

^c^ We considered sulbactam a beta-lactam in this context, due to its intrinsic antibacterial activity against *Acinetobacter.*

MIC_50_ underlined, MIC_90_ in bold.

**Table 3:** Minimum inhibitory concentrations and resistance rates of *Acinetobacter baumannii* isolates, Spain, 2020 (n = 118)

Class of antibiotic	Per cent of isolates at MIC (mg/L)												% R
	≤ 0.12	0.25	0.5	1	2	4	8	16	32	64	128	≥ 256	
Beta-lactams													
IMI	ND	36.4^a^	41.5	42.4	42.4	43.2	51.7	65.2	**96.6**	100^b^	ND	ND	56.8
MEM	ND	19.5^a^	35.6	42.4	42.4	42.4	46.6	53.4	66.9	**100^b^**	ND	ND	57.6
FEP	ND	ND	1.7^a^	19.5	34.7	39.8	41.5	50.0	76.3	88.1	**100^b^**	ND	50.0
FDC	75.4	**91.5**	96.6	98.3	100	ND	ND	ND	ND	ND	ND	ND	0.0
SUL^c^	ND	0.0^a^	31.4	39.8	47.6	56.8	83.9	**95.8**	99.1	100^b^	ND	ND	16.1
Aminoglycosides													
AMK	ND	ND	ND	5.9^a^	17.8	53.4	61.9	67.8	69.5	71.2	72.0	**100**	30.5
TOB	ND	ND	ND	56.8^a^	57.6	61.9	62.7	66.9	67.8	70.3	76.3	**100**	37.3
Fluoroquinolones													
CIP	ND	36.4^a^	39.8	39.8	40.7	40.7	42.4	43.2	52.5	**100** ^b^	ND	ND	59.3
Polymyxins													
COL	0.8	5.9	23.7	58.5	86.4	**94.1**	99.1	99.1	99.1	99.1	100^b^	ND	13.6

AMK: amikacin; CIP: ciprofloxacin; COL: colistin; FDC: cefiderocol; FEP: cefepime; IMI: imipenem; MEM: meropenem; MIC: minimum inhibitory concentration; ND: not determined; R: resistance; SUL: sulbactam; TOB: tobramycin.

^a^ Actual MIC value is equal or inferior to that stated.

^b^ Actual MIC value is equal or higher to that stated.

^c^ We considered sulbactam a beta-lactam in this context, due to its intrinsic antibacterial activity against *Acinetobacter.*

MIC_50_ underlined, MIC_90_ in bold.

The susceptibility rates among *A.* non-*baumannii* species, provided in Supplementary Table S2 **,** ranged from 97.5 to 100% for all tested antimicrobials, excluding colistin, to which 9.9% of isolates were resistant. The MIC values and genetic characteristics for each *A.* non-*baumannii* isolate can be found in Supplementary Table S4. These colistin-resistant strains (*A. dispersus *(n = 1), *Acinetobacter* spp. (n = 2), *A. bereziniae* (n = 2), *A. beijerinckii* (n = 1), *A. colistiniresistens* (n = 1) and *A. haemolyticus* (n = 1)) displayed MICs ranging from 4 to 32 mg/L.

**Figure 2:**
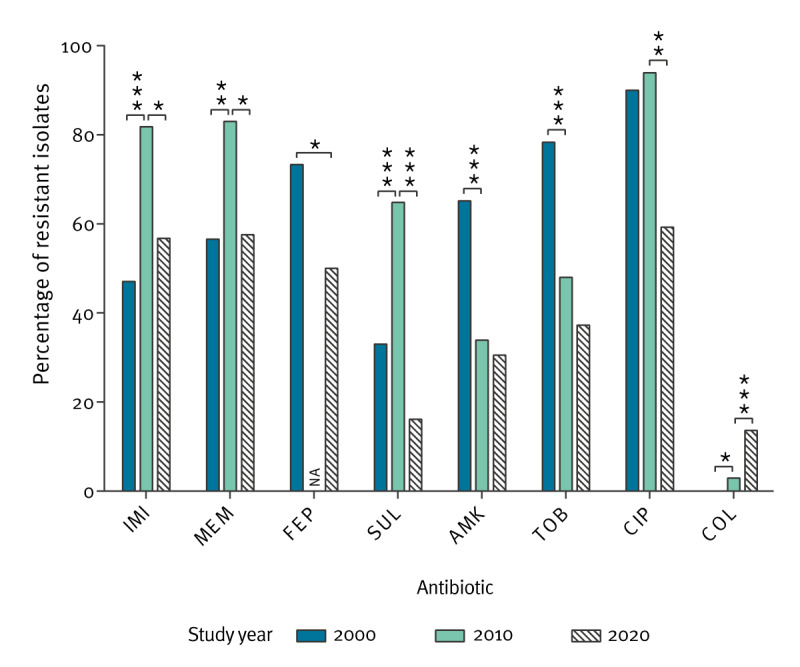
Differences between resistance rates of *Acinetobacter baumannii* clinical strains from the 2000 [12,13], 2010 [14,15] and 2020 *Acinetobacter* species collections, Spain

AMK: amikacin; CIP: ciprofloxacin; COL: colistin; FEP: cefepime; IMI: imipenem; MEM: meropenem; MIC: minimum inhibitory concentration; NA: data not available; SUL: sulbactam; TOB: tobramycin.

Changes in antimicrobial resistance rates were compared to the two prior Spanish *A. baumannii* surveys, conducted in 2000 and 2010 [12-15] (Figure 2). Of the carbapenems, resistance rates of both meropenem and imipenem increased substantially between 2000 and 2010. However, upon analysing the data from our *A. baumannii* collection, a clear decrease in these parameters became apparent from 2010 onwards (p = 0.0295 for imipenem and p = 0.0293 for meropenem), reaching a resistance rate of around 60%. Variations in resistance followed a fluctuating pattern for sulbactam. Aminoglycosides displayed a gradual decline in resistance across the three study periods. Nonetheless, it is worth noting that the aminoglycoside data from 2000 may be somewhat misleading because of the grouping of intermediate and resistant isolates, owing to the lack of MIC distributions. The proportion of ciprofloxacin-susceptible isolates has also increased since 2010. Lastly, a troubling and highly significant surge in colistin resistance was observed between 2000 (0%) and 2020 (13.6%) (Figure 2, Table 3).

Resistance rates for 2000 AMK and TOB include intermediate isolates (MIC = 32 mg/L and MIC = 8 mg/L, respectively) because of the unavailability of MIC distribution data for that period.

Cefiderocol was not included as it was not an available antibiotic in 2000 and 2010.

Statistically significant shifts are marked by asterisks as follows: p < 0.05 *, p < 0.01 **, p < 0.001 ***.

### Phylogeny and molecular epidemiology of *Acinetobacter* species

**Figure 3:**
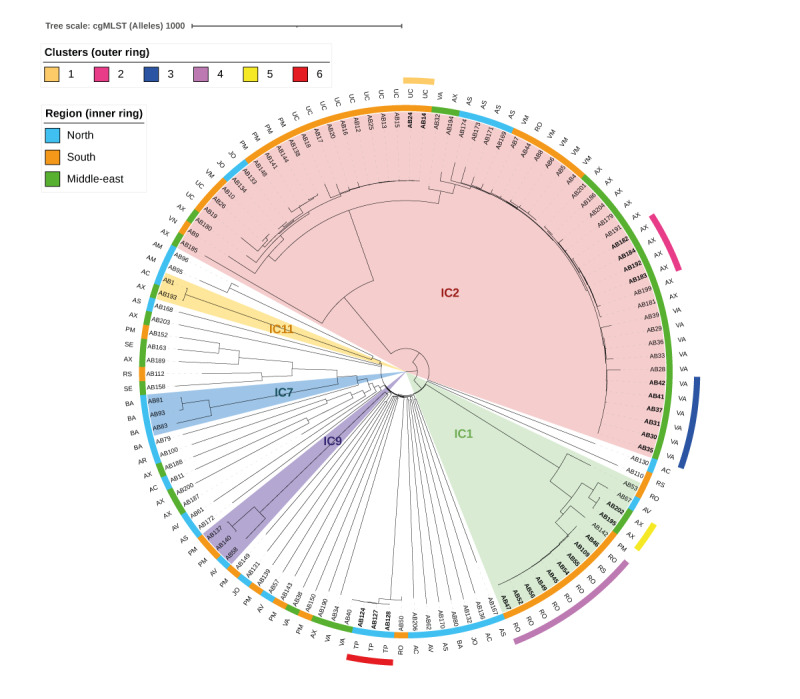
Phylogenetic distribution of *Acinetobacter baumannii* isolates, Spain, 2020 (n = 118)

Core genome multilocus sequence typing (cgMLST) and phylogenetic analysis were conducted on the *A. baumannii* isolates to discern clonal lineages or regional differences in epidemiology in Spain (Figure 3). Currently, 11 international clones (IC) are acknowledged for *A. baumannii*; our study identified five among our 118 isolates: IC1 (ST1), IC2 (ST2, ST745), IC7 (ST25), IC9 (ST6, ST85) and IC11 (ST164). Within this set, IC2 emerged as the most prevalent and widely distributed in Spain, followed by IC1. Hence, clear predominance of ST2 (n = 52 isolates, 44.1%) and ST1 (n = 14 isolates, 11.8%) was observed. The remaining 52 strains (44.1%) represented 35 different STs (Pasteur scheme), thus highlighting an extensive clonal dispersion beyond the ST1 and ST2 lineages across Spain.

AC: A Coruña University Hospital; VM: University Hospital Virgen Macarena; VN: University Hospital Virgen de las Nieves; UC: Canary Islands University Hospital; VA: University Clinical Hospital of Valencia; RO: University Hospital Virgen del Rocío; AV: University Hospital Arnau de Vilanova; BA: Basurto University Hospital; AM Hospital Arquitecto Marcide; AR: Araba University Hospital; RS: University Hospital Reina Sofía; TP: University Hospital Germans Trias i Pujol; JO: University Hospital Joan XXIII; PM: University Hospital Puerta del Mar; SE: University Hospital Son Espases; AS: Asturias Central University Hospital; AX: University Clinical Hospital Virgen de la Arrixaca.

The phylogeny is based on cgMLST. The outer ring illustrates the clonal relationships among isolates; those sharing a clonal relationship (cgMLST ≤ 3 SNPs) are visually linked by the same colour within their respective clonal clusters. The middle ring displays the abbreviations of the hospitals from which the isolates were obtained. The inner ring provides a geographical context by representing the regions where the respective hospitals are situated. The shaded portions in the inner circle represent the international clone grouping to which the isolates belong.

Six clusters of isolates, exhibiting closely related genomes (≤ 3 cgMLST allele distance), were identified, suggesting clonal dissemination. Notably, a substantial portion of ST1 (IC1) comprised clonal isolates, specifically within Clusters 4 and 5. Interhospital clonal transmission was also detected within Cluster 4 (Figure 3).

*Acinetobacter baumannii* displayed some differences regarding its distribution between different Spanish regions. ST2 (IC2) was predominantly isolated in the southern and middle-eastern regions, while ST1 (IC1) prevailed in the south because of clonal transmission. The northern regions exhibited higher epidemiological heterogeneity in *A. baumannii*.

In contrast, the phylogenetic analysis of the most abundant *A.* non-*baumannii* species, *A. pittii* (n = 28) and *A. ursingii* (n = 13), emphasised their heightened prevalence in northern regions. The phylogenetic tree for *A.* non-*baumannii* is provided in Supplementary Figure S1. Probable intrahospital dissemination (< 0.0000001 branch length) was observed, notably involving isolates API162 and API160 from *A. pittii*, as well as AU166 and AU165 from *A. ursingii*. However, an overall pattern of phylogenetic heterogeneity was observed within these two *A.* non-*baumannii* species.

### Antimicrobial resistance mechanisms across *Acinetobacter* species

Sequence types intricately link to the resistome. Of the ST2 isolates, 80.8% (42/52) were not susceptible to at least three antimicrobial families (multidrug-resistant (MDR) isolates), with 61.5% (32/52) classified as extensively drug-resistant (XDR). Further, four of the 14 ST1 isolates were also categorised as MDR. Other *A. baumannii* STs exhibited overall higher rates of susceptibility to the antibiotics tested.

The key phenotypic and genotypic characteristics of antimicrobial resistance in the *A. baumannii* isolates are listed in Supplementary Table S3. Predominantly, carbapenem resistance stemmed from carbapenem-hydrolysing-class D beta-lactamases (CHDLs), particularly OXA-23, detected in 49 strains (41.5%). OXA-58 and OXA-24/40 were produced in eight (6.8%) and one (0.8%) isolate, respectively. ST2 strains mainly carried OXA-23 (69.2%, 36/52), driving its prevalence in Spain. Furthermore, OXA-58 appeared in four ST2 and four ST745 isolates. All CHDL-producing strains exhibited high imipenem and meropenem MICs (≥ 8 mg/L), being resistant to both. Non-CHDL-producing strains also displayed some resistance to carbapenems. Ten ST2 isolates producing only OXA-201, an OXA-51-like oxacillinase, showed varied carbapenem resistance (4–32mg/L) because of the presence of an IS*Aba*1 insertion sequence upstream of the gene. IS*Aba*1 and other insertion sequences can notably enhance the expression of multiple genes in *A. baumannii* [23]. Accordingly, IS*Aba*1 was also localised upstream of the *bla*_OXA-23_, or *bla*_ADC-like_ genes in numerous ST2 isolates (n = 22).

Among the 68 carbapenem-resistant *A. baumannii* strains, six also showed porin modifications: three with Omp33–36 insertions, two with incomplete OprD proteins and one with an incomplete CarO (Supplementary Table S3). Two *A.* non-*baumannii* strains, one *A. nosocomialis* and one *A. junii*, both from the same hospital, displayed carbapenem resistance. The MIC values and genetic characteristics for each *A.* non-*baumannii* isolate are included in Supplementary Table S4. A carbapenem-susceptible strain of *A. radioresistens* produced OXA-23. No cefiderocol resistance or reduced susceptibility was noted. However, 50.0% of the *A. baumannii* isolates were resistant to cefepime, and 16.1% to sulbactam. Cefepime resistance was predominantly related to the presence of a CHDL or the insertion of IS*Aba*1 in the OXA-51-like promoter. Similarly, higher sulbactam MICs were associated with isolates carrying OXA-23, while moderate MICs were detected in IS*Aba*1/OXA-51-like producing isolates. Four isolates with MICs ≥ 8 mg/L to sulbactam also carried the IS*Aba*1/ADC-30 combination (Supplementary Table S3).

Among the highly resistant ST2 isolates to tobramycin and/or amikacin (n = 24), the ArmA methylase and the AAC(6’)-Ib7 acetyltransferase were present in 95.8% (23/24) and 79.2% (19/24), respectively. Additionally, ANT(2”)-Ia (2″-O-nucleotidyltransferase) and APH(3')-VIa (phosphotransferase) were highly prevalent in their ST1 homologues. Susceptible strains either lacked aminoglycoside-modifying enzymes or possessed a smaller genetic arsenal (Supplementary Table S3). As for the *A.* non-*baumannii* strains, ca 21% exhibited aminoglycoside-modifying enzymes, primarily AAC-types (Supplementary Table S4).

This study revealed an increase in colistin resistance among carbapenem-resistant *A. baumannii* isolates in Spain, reaching 13.6% in 2020. Whole genome sequencing unveiled mutations (Q34P and L94M in PmrB and A46T in PmrC) in three colistin-resistant strains (Supplementary Table S3). Of note, the absence of *mcr* genes paralleled the lack of insertion sequences upstream of the *pmrCAB* genes. The conventional LPS modification mechanism through PmrCAB, responsible for colistin resistance, was also absent in some strains within our collection. Hence, the specific resistance mechanism in these isolates remains to be clarified. Among *A.* non*-baumannii*, eight isolates (9.9%) displayed colistin resistance, which is a relatively high level of resistance to this antibiotic. Multiple mutations in PmrC and PmrB were identified (Supplementary Table S4). However, the scarce amount of literature regarding the mechanisms of colistin resistance and the lack of reference genomes for these species prevent us from precisely assessing the possible genes involved in these *A.* non-*baumannii* isolates.

## Discussion

*Acinetobacter* spp. prevalence within Spanish hospitals has shown a notable decline from around 2010 onwards. Studies on the occurrence of healthcare-associated infections indicate a decreasing trend in *A. baumannii*’s involvement in nosocomial infections, dropping from 2.7% in 2011 to 0.43% in 2021 and further to 0.23% in 2022 [24]. This situation may be shared by neighbouring countries, as observed in several European Centre for Disease Prevention and Control (ECDC) point prevalence surveys (PPS) assessing healthcare-associated infections in acute care hospitals; the incidence of *Acinetobacter* spp. infections decreased between the periods 2011–12 and 2016–17 in Portugal (from 6.5% to 1.1%), France (from 2% to 1.4%) and Italy (from 5.7% to 3.1%) [25,26].

Despite the decline in *A. baumannii*, its importance persists because of the complex and challenging nature of the resulting infections. The proportion of *A. baumannii*-derived infections in Spain has seemingly increased over the past 20 years (52.9%, 61.4% and 73.7% in 2000, 2010 and 2020, respectively), independent of the overall presence of this bacterium in hospital settings. Colonisation rates showed a contrasting trend (47.1% in 2000, 38.7% in 2010 and 20.1% in 2020) [15]. However, neither of these trends proved to be statistically significant in our study.

Regarding infection types, epidemiological data from the ECDC 2016–17 PPS highlighted that in Europe, *Acinetobacter* spp. primarily causes respiratory and bloodstream infections [26]. These data align with the predominant infection patterns observed in Spain during 2000 and 2010, with respiratory infections being the most prevalent [13,15]. However, our observations indicate a shift in this trend, as SSTIs emerged as the main outcome associated with *A. baumannii* in 2020.

As observed, antimicrobial resistance trends showcased a significant increase in colistin resistance accompanied by a decrease in carbapenem resistance in 2020. Information from the previous multicentre studies also indicates a shift in treatment patterns for *A. baumannii* infections. Carbapenems were predominantly used in 2000, while the preference shifted to colistin by 2010, likely influenced, as Villar et al. noticed, by the escalating carbapenem resistance during that period [15]. Hence, acknowledging the intricate interplay between antibiotic consumption and resistance is important, considering their substantial impact over time. According to surveillance data from the ECDC survey of antibiotic usage in Spain [27], general use of these compounds decreased by 7.3% in 2019 (relative to the average value of preceding years, commencing from 2016), and by 20.6% between 2019 and 2020. Detailed analysis by antimicrobial group showed that this tendency was shared by both carbapenems (18.2% reduction between 2016 and 2020) and fluoroquinolones (38.8% reduction between 2016 and 2020). For polymyxins, use of these compounds in Spain, even though it is still lower than that of other antibiotics, has increased gradually since the first available records in 2016 (41.3%), and interestingly, by 2020 the use of polymyxins remained 4.7-fold higher than the average for the European Union [27]. While these findings may correlate with the changing trends observed for *A. baumannii* in terms of antimicrobial resistance, specific treatment guidelines for each pathogen will most likely play a major role. A recent study on *P. aeruginosa* detected significant decreases in carbapenem, ciprofloxacin and colistin resistance rates between 2017 and 2022 [28], whereas for *E. coli*, ECDC reported diminishing resistance to fluoroquinolones (32.8% in 2016 and 28.6% in 2020) but not for carbapenems (0.1% in 2016 and 0.4% in 2020) [29].

High non-susceptibility rates were still observed in our 2020 *Acinetobacter* spp. collection, specifically in *A. baumannii*. In a similar study in Germany, with specimens collected between 2010 and 2019 [30], Wohlfarth et al. found that in 2019 around 15%, 25%, 20% and 1% of isolates were resistant to aminoglycosides, ciprofloxacin, imipenem/meropenem and colistin, respectively, using EUCAST breakpoints. However, when applying the same guidelines, resistance rates were clearly higher in our collection of isolates (around 40% for aminoglycosides, 60% for ciprofloxacin, 55% for imipenem/meropenem and 14% for colistin as provided in Supplementary Table S5), highlighting regional differences in Europe. Conversely, Karlowsky et al. revealed similar profiles (4,038 global *A. baumannii* isolates from 2016 to 2021, using CLSI guidelines) to those we observed [31]. Major differences were found in sulbactam and colistin resistance rates, which were around threefold higher for sulbactam (54.6%) and at least twofold lower for colistin (4.9%) than in our collection (16.1% and 13.6%, respectively).

Data on the currently circulating STs within Spain were consistent with that of the global epidemiology of *A. baumannii*. Worldwide, ST1 and ST2 are the most successfully spread, commonly harbouring the acquired carbapenemase OXA-23 [32,33]. In this context, the selection and dissemination of difficult-to-treat isolates, largely associated with these two major sequence types, raise notable concerns [34].

In comparison to the data from 2000 to 2010, the predominant sequence types during that period were ST2 (52.9%), ST79 (10.7%), ST181 (7.9%) and ST179 (3%) [15]. Between 2000 and 2010, ST2 isolates in Spanish hospitals rose from 42.9% to 59%, while ST79 and ST81 percentages remained relatively unchanged. Despite ST2 maintaining its predominant status in 2020, its prevalence dropped by ca 15% in a decade, and ST1 has emerged as the second most prevalent sequence type. Interestingly, no ST79, ST181 or ST179 isolates were observed in 2020. On the contrary, the majority of the identified STs (94.6%) were minor ones (≤ 5 patients), mirroring the findings from 2000 and 2010. This suggests a sustained pattern of *A. baumannii*’s clonal dispersion over the course of 2 decades [13,15].

Regarding international clones, the displacement of ST79 (IC5) by ST1 (IC1) as the second most prevalent clone, alongside the presence of sporadic ICs (IC7, IC9, IC11), suggests shared evolutionary connections extending beyond national boundaries. French isolates from 2010 to 2011 showcased the presence of IC1, IC7 and IC9 (all in comparable proportions), secondary to the predominant IC, IC2 [35]. Notably, the currently absent IC5 had minimal presence during that period in France. Additionally, IC7 and IC9 exhibited anecdotal prevalence among CRAB strains in Germany in 2016, contrasting with their prior absence [30]. This pattern seemingly aligns with their limited contribution to present-day carbapenem resistance in Spain.

The events of intrahospital and, in one case, interhospital clonal transmission spanning IC2 and IC1 isolates in our study, highly associated with multidrug resistance, are concerning. This underscores the need for robust infection prevention and control (IPC) measures to halt their potential nosocomial dissemination.

 Although the impact of the mutations in porins such as CarO, Omp33-36, and OprD on carbapenem-resistant isolates was not assessed, they do not appear to significantly influence high carbapenem MIC values [36]. Instead, in this study, OXA-23 stands out as the most prevalent carbapenem resistance mechanism among *A. baumannii*. Analysis within the 2000 and 2010 studies highlighted important changes in the epidemiology of CHDLs in Spain. Thus, *bla*_OXA-24/40-like_, *bla*_OXA-58-like_ and the IS*Aba*1-*bla*_OXA-51-like_ combination were identified in 48.7%, 20.5% and 23% of the isolates from 2000, and in 51.6%, 34.4% and 19.6% of the isolates from 2010. Notably, the acquisition of the *bla*_OXA-24/40_ gene was the main driver of carbapenem resistance in 2000 and 2010 [37]. This carbapenemase was mostly detected in ST2 *A. baumannii* strains in both 2000 and 2010, although STs such as ST79 and ST80 also produced OXA-24/40 in 2010. Of note, the *bla*_OXA-23_ gene was only found in one strain in the GEIH/REIPI-Ab-2010 study. Therefore, the data contrast with that obtained by WGS for the *A. baumannii* isolates retrieved in 2020, revealing a shift in the epidemiology of CHDLs in Spain over the last decade. The OXA-24/40 carbapenemase has been displaced by OXA-23, while being also carried by ST2 isolates [38,39].

Higher sulbactam MICs have also been associated to the presence of particular beta-lactamases in our collection. As observed, carbapenemases such as OXA-23, or the cephalosporinase ADC-30 have been proved to impact sulbactam activity in the *A. baumannii* background [5,32,40]. 

Two *A.* non-*baumannii* isolates, *A. nosocomialis* and *A. junii*, exhibited carbapenem resistance attributed to the presence of OXA-24/40. This has been documented previously within this strain collection [41], elucidating the dissemination of OXA-24/40 facilitated by small GR12 plasmids among *Acinetobacter* species. Additionally, one carbapenem-susceptible *A. radioresistens* isolate was found to produce OXA-23. This phenomenon*,* previously reported in *A. radioresistens*, denotes a silent, low expression source of this carbapenemase [42]. According to our findings, colistin resistance has increased in the past 20 years, and this proportion is higher than that in worldwide surveillance [30-32]. This pattern has been observed in many other recent studies, underscoring the lack of awareness despite the mounting prevalence of colistin-resistant isolates [32,43]. Although the publication of colistin-resistant *A. baumannii* isolates is becoming increasingly frequent, the mechanism of resistance to this antibiotic in *A. baumannii* is rarely described. Thus, while conventional mechanisms could account for resistance in some strains, the resistance in many others remained unexplained.

The expansion in recent decades of clinical isolates of *A. baumannii* that are resistant to carbapenems and to most available antibiotics is a clinical problem of concern. There is an apparent dominance of a few successful lineages, whose mechanism of spread warrants detailed study. The presence of OXA-23 in easily spreadable mobile elements, such as the Tn2006 transposon, favours the presence and expansion of these high-risk-clones in the hospital environment. Studies such as the present national level study are crucial to obtain a complete and up-to-date picture of the dissemination of these clones and their mechanisms of resistance in order to apply appropriate interventions [33].

Our study has some limitations. While 18 hospitals participated, incomplete representation of some Spanish regions resulted from either unresponsive facilities or the absence of reported *Acinetobacter* spp. during the collection period. In addition, the limited number of isolates could be attributed to effective sanitary controls in Spain, leading to a decreased occurrence of *Acinetobacter* spp. in healthcare facilities. 

## Conclusions

Our study highlights the relevance of continuous epidemiological surveillance, accurate identification and understanding of antibiotic resistance patterns among *Acinetobacter* spp. Notably, *A.* non-*baumannii* species constitute nearly half of the cases, yet demonstrate susceptibility to most tested antibiotics compared to *A. baumannii*. The shift in CRAB epidemiology observed, marked by the decline of OXA-24/40 and the ascent of OXA-23-producing ST2 and ST1, mirrors trends in neighbouring European countries. Furthermore, except for colistin, overall resistance rates have either stabilised or decreased over the past decade. This knowledge is fundamental for steering healthcare policies, developing effective treatment guidelines, and implementing infection control strategies to diminish the impact of these infections on public health.

## Supplementary Data

569000 Supplement
