# Supplementary material for: Epidemiology, resistance genomics and susceptibility of Acinetobacter species: results from the 2020 Spanish nationwide surveillance study
Source: Euro Surveill. 2024 Apr 11;29(15):2300352. doi: 10.2807/1560-7917.ES.2024.29.15.2300352 (PMC11010588; doi:10.2807/1560-7917.ES.2024.29.15.2300352)
Supplement: Supplement [file 23-00352_VAZQUEZ_UCHA_Supplement.pdf]

SUPPLEMENTARY MATERIAL

Title: Epidemiology, resistance genomics and susceptibility of *Acinetobacter* species: results from the 2020 Spanish nationwide surveillance study

This supplementary material is hosted by Eurosurveillance as supporting information alongside the article [Epidemiology, resistance genomics and susceptibility of *Acinetobacter* species: results from the 2020 Spanish nationwide surveillance study], on behalf of the authors, who remain responsible for the accuracy and appropriateness of the content. The same standards for ethics, copyright, attributions, and permissions as for the article apply. Supplements are not edited by Eurosurveillance, and the journal is not responsible for the maintenance of any links or email addresses provided therein.

TABLE S1……………………………………………………………………..2

TABLE S2……………………………………………………………………..3

TABLE S3……………………………………………………………………10

TABLE S4……………………………………………………………………15

TABLE S5……………………………………………………………………16

FIGURE S1…………………………………………………………………..17

Materials and Methods………………………………………………………18

References …………………………………………………………………..19

Table S1. Origin of the isolates and their relative distribution per hospital, Spain, 2020 (n=199)

Region	Site	Isolates (n)		
		Infection	Colonisation	TOTAL
N	A Coruna University Hospital	13	2	**15**
S	University Hospital Virgen Macarena	3	3	**6**
S	University Hospital Virgen de las Nieves	1	0	**1**
S	Canary Islands University Hospital	8	4	**12**
M-E	University Clinical Hospital of Valencia	14	1	**15**
S	University Hospital Virgen del Rocío	10	1	**11**
N	University Hospital Arnau de Vilanova	13	8	**21**
N	Basurto University Hospital	17	0	**17**
N	Hospital Arquitecto Marcide	6	0	**6**
N	Araba University Hospital	8	0	**8**
S	University Hospital Reina Sofía	14	1	**15**
N	University Hospital Germans Trias I Pujol	6	0	**6**
N	University Hospital Joan XXIII	3	1	**4**
S	University Hospital Puerta del Mar	10	7	**17**
M-E	University Hospital Son Espases	11	0	**11**
N	Asturias Central University Hospital	2	6	**8**
M-E	University Clinical Hospital Virgen de la Arrixaca	19	6	**25**
N	Naval Hospital Ferrol	1	0	**1**
	**ALL**	**159**	**40**	**199**

Abbreviations: N (North), S (South), Middle-east (M-E)

Table S2. MIC distributions and resistance rates to the antibiotics tested relative to the set of *A.* non-*baumannii* isolates, Spain, 2020 (n= 81).

Class of antibiotic	Per cent of isolates at MIC (mg/L)												% R
	≤0.12	0.25	0.5	1	2	4	8	16	32	64	128	≥256	
**Beta-lactams**													
IMI		**<u>92.6</u>**[a]	97.5	97.5	97.5	97.5	97.5	97.5	100				2.5
MEM		44.4[a]	<u>87.6</u>	**97.5**	97.5	97.5	97.5	97.5	97.5	100[b]			2.5
FEP			12.3[a]	33.3	<u>75.3</u>	**91.4**	97.5	100					0.0
FDC	<u>62.7</u>	79.0	86.4	**96.3**	100								0.0
SUL[c]		11.1[a]	<u>80.2</u>	**95.1**	100								0.0
**Aminoglycosides**													
AMK				28.4[a]	<u>55.6</u>	88.9	**96.3**	98.8	100				0.0
TOB				<u>85.2</u>[a]	**92.6**	96.3	98.8	98.8	98.8	100			1.2
**Fluoroquinolones**													
CIP		**<u>90.1</u>**[a]	92.6	96.3	97.5	97.5	97.5	97.5	97.5	100[b]			2.5
**Polymixins**													
COL	6.2	13.6	46.9	<u>74.1</u>	**90.1**	95.1	97.5	98.8	100				9.9

AMK: amikacin; CIP: ciprofloxacin; COL: colistin; FDC: cefiderocol; FEP: cefepime; IMI: imipenem; MEM: meropenem; MIC: minimum inhibitory concentration; R: resistance; SUL: sulbactam; TOB: tobramycin.

[a] Actual MIC value is equal or inferior to that stated.

[b] Actual MIC value is equal or higher to that stated.

[c] We considered sulbactam a beta-lactam in this context, due to its intrinsic antibacterial activity against *Acinetobacter.*

MIC_{50} underlined, MIC_{90} in bold.

Table S3. Species identification, isolation site, ST, MIC values and putative resistance mechanisms for the *A. baumannii* isolates, Spain, 2020 (n=118).

ID	Species	Site	ST	MIC (mg/L)									Putative resistance mechanisms[a]			
				IMI	MEM	FEP	FDC	SUL	AMK	TOB	CIP	COL	beta-lactamases	AMEs and 16S rRNA methyltrans-ferases	Porins and PBPs	PmrCAB and related systems
1	*A. baumannii*	AC	164	≤0.25	0.5	2	≤0.12	0.5	4	≤1	≤0.25	1	ADC-6, OXA-91		OprD D421insFN	
4	*A. baumannii*	VM	2	32	≥64	32	≤0.12	8	4	16	≥64	1	ADC-30, OXA-66, IS*Aba1*-OXA-23	AAC(6')-Ib7, ANT(3'')-IIa, APH(3')-Ia, APH(3'')-Ib, APH(6)-Id, ArmA		
5	*A. baumannii*	VM	2	32	≥64	32	≤0.12	8	4	16	≥64	0.5	ADC-30, OXA-66, IS*Aba1*-OXA-23	AAC(6')-Ib7, ANT(3'')-IIa, APH(3'')-Ib, APH(6)-Id, ArmA		
6	*A. baumannii*	VM	2	16	≥64	32	≤0.12	8	2	16	≥64	0.5	ADC-30, OXA-66, IS*Aba1*-OXA-23, TEM-12	AAC(6')-Ib7, APH(3')-Ia, APH(3'')-Ib, ArmA		
7	*A. baumannii*	VM	2	16	32	≥128	2	16	8	2	≥64	1	ADC-30, OXA-66, IS*Aba1*-OXA-23,	APH(3'')-Ib, APH(6)-Id		
8	*A. baumannii*	VM	2	32	≥64	16	≤0.12	8	4	≤1	≥64	1	ADC-30, OXA-66, IS*Aba1*-OXA-23,	APH(3'')-Ib, APH(6)-Id		
9	*A. baumannii*	VN	2	8	16	32	0.25	16	≥256	≥256	≥64	2	ADC-30, OXA-66, OXA-23, TEM-12	AAC(3)-Ia, APH(3')-Ia, APH(3'')-Ib, APH(6)-Id, ArmA		
10	*A. baumannii*	VM	2	32	≥64	32	0.5	8	4	16	≥64	0.5	ADC-30, OXA-66, IS*Aba1*-OXA-23	AAC(6')-Ib7, APH(3'')-Ib, APH(6)-Id		
11	*A. baumannii*	AC	239	≤0.25	1	2	0.25	4	2	≤1	2	2	ADC-2, OXA-51			
12	*A. baumannii*	UC	2	8	32	16	0.5	2	4	≤1	≥64	2	ADC-30, IS*Aba1*-OXA-201	AAC(3)-Ia, ANT(3'')-IIa, APH(3')-Ia		
13	*A. baumannii*	UC	2	8	32	16	0.25	4	4	≤1	≥64	2	ADC-30, IS*Aba1*-OXA-201	AAC(3)-Ia, ANT(3'')-IIa, APH(3')-Ia		
14	*A. baumannii*	UC	2	16	32	16	0.25	2	2	≤1	≥64	2	ADC-30, IS*Aba1*-OXA-201	AAC(3)-Ia, ANT(3'')-IIa, APH(3')-Ia		
15	*A. baumannii*	UC	2	8	32	≥128	≤0.12	4	≤1	≤1	≥64	2	ADC-30, IS*Aba1*-OXA-201	AAC(3)-Ia, ANT(3'')-IIa		
16	*A. baumannii*	UC	2	8	32	32	≤0.12	2	4	≤1	≥64	2	ADC-30, IS*Aba1*-OXA-201	AAC(3)-Ia, ANT(3'')-IIa		
17	*A. baumannii*	UC	2	8	16	32	0.25	2	2	≤1	≥64	4	ADC-30, IS*Aba1*-OXA-201	AAC(3)-Ia, ANT(3'')-IIa, APH(3')-Ia		
18	*A. baumannii*	UC	2	8	32	32	≤0.12	2	4	≤1	≥64	4	ADC-30, IS*Aba1*-OXA-201	AAC(3)-Ia, ANT(3'')-IIa, APH(3')-Ia		

19	*A. baumannii*	UC	2	16	16	64	≤0.12	4	16	64	≥64	4	ADC-30, OXA-66, OXA-58	AAC(3)-IIe, APH(6)-Id, APH(3'')-Ib	
20	*A. baumannii*	UC	2	8	16	16	1	2	4	≤1	32	2	ADC-30, IS*Aba1*-OXA-201	AAC(3)-Ia, ANT(3'')-IIa, APH(3')-Ia	
24	*A. baumannii*	UC	2	4	32	16	0.25	4	8	4	≥64	2	ADC-30, IS*Aba1*-OXA-201	AAC(3)-Ia, ANT(3'')-IIa, APH(3')-Ia	
25	*A. baumannii*	UC	2	8	32	32	≤0.12	4	4	≤1	≥64	2	ADC-30, IS*Aba1*-OXA-201	AAC(3)-Ia, ANT(3'')-IIa, APH(3')-Ia	
26	*A. baumannii*	UC	2	16	16	64	≤0.12	4	16	32	≥64	4	ADC-30, OXA-66, OXA-58	AAC(3)-IIe, APH(3'')-Ib, APH(6)-Id	PmrB Q34P
28	*A. baumannii*	VA	2	32	≥64	64	0.5	8	≥256	≥256	≥64	8	IS*Aba1*-ADC-30, OXA-66, IS*Aba1*-OXA-23	AAC(6')-Ib7, ANT(3'')-IIa, APH(3'')-Ib, APH(6)-Id, ArmA	
29	*A. baumannii*	VA	2	32	≥64	64	≤0.12	8	4	≤1	≥64	2	ADC-30, OXA-66, OXA-23	APH(3'')-Ib, APH(6)-Id	
30	*A. baumannii*	VA	2	32	≥64	32	0.25	8	≥256	≥256	≥64	8	IS*Aba1*-ADC-30, OXA-66, IS*Aba1*-OXA-23	AAC(6')-Ib7, ANT(3'')-IIa, APH(3'')-Ib, APH(6)-Id, ArmA	
31	*A. baumannii*	VA	2	32	≥64	32	≤0.12	16	4	≤1	≥64	2	ADC-30, OXA-66, IS*Aba1*-OXA-23	APH(3'')-Ib, APH(6)-Id	
32	*A. baumannii*	VA	2	32	≥64	32	0.25	8	≥256	≥256	≥64	8	IS*Aba1*-ADC-30, OXA-66, IS*Aba1*-OXA-23	ANT(3'')-IIa, APH(3'')-Ib, APH(6)-Id, ArmA	
33	*A. baumannii*	VA	2	32	≥64	64	≤0.12	16	4	≤1	≥64	1	ADC-30, OXA-66, IS*Aba1*-OXA-23	APH(3'')-Ib, APH(6)-Id	
34	*A. baumannii*	VA	2182	≤0.25	≤0.25	2	≤0.12	0.5	4	≤1	≤0.25	0.5	ADC-2, OXA-130		
35	*A. baumannii*	VA	2	32	≥64	32	0.25	8	≥256	≥256	≥64	8	ADC-30, OXA-66, IS*Aba1*-OXA-23	AAC(6')-Ib7, ANT(3'')-IIa, APH(3'')-Ib, APH(6)-Id, ArmA	
36	*A. baumannii*	VA	2	32	≥64	32	≤0.12	8	4	≤1	≥64	1	ADC-30, OXA-66, IS*Aba1*-OXA-23	APH(3'')-Ib, APH(6)-Id	
37	*A. baumannii*	VA	2	32	≥64	32	0.25	8	≥256	≥256	≥64	8	ADC-30, OXA-66, IS*Aba1*-OXA-23	AAC(6')-Ib7, ANT(3'')-IIa, APH(3'')-Ib, APH(6)-Id, ArmA	
38	*A. baumannii*	VA	866	≤0.25	0.5	4	≤0.12	0.5	32	≥256	≤0.25	2	ADC-2, OXA-385	ANT(2'')-Ia	
39	*A. baumannii*	VA	2	8	8	2	≤0.12	4	≥256	≥256	≥64	2	ADC-30, OXA-66, OXA-23	AAC(6')-Ib7, ANT(3'')-IIa, APH(3'')-Ib, APH(6)-Id, ArmA	
40	*A. baumannii*	VA	2176	≤0.25	≤0.25	≤0.5	≤0.12	0.5	4	≤1	≤0.25	1	ADC-2, OXA-64		
41	*A. baumannii*	VA	2	32	≥64	32	0.25	8	≥256	≥256	≥64	4	ADC-30, OXA-66, IS*Aba1*-OXA-23	AAC(6')-Ib7, ANT(3'')-IIa, APH(3'')-Ib, APH(6)-Id, ArmA	

42	*A. baumannii*	VA	2	32	32	32	≤0.12	8	≥256	≥256	≥64	8	ADC-30, OXA-66, IS*Aba1*-OXA-23	AAC(6')-Ib7, ANT(3")-IIa, APH(3")-Ib, APH(6)-Id, ArmA	
44	*A. baumannii*	RO	2	16	32	16	≤0.12	4	16	64	≥64	0.25	ADC-30, OXA-66, OXA-23	AAC(6')-Ib7, ANT(3")-IIa, APH(3')-Ia, APH(3")-Ib, ArmA	
45	*A. baumannii*	RO	1	32	≥64	64	≤0.12	16	≥256	128	32	2	ADC-75, OXA-69, OXA-23	ANT(2")-Ia, APH(3')-Ia	
46	*A. baumannii*	RO	1	32	≥64	64	≤0.12	16	≥256	128	≥64	1	ADC-75, OXA-69, OXA-23	ANT(2")-Ia, APH(3')-Ia	
47	*A. baumannii*	RO	1	32	≥64	64	≤0.12	16	≥256	128	≥64	1	ADC-75, OXA-69, OXA-23	ANT(2")-Ia, APH(3')-Ia, APH(3')-VIa	Omp33-36 ΔV43insT& A45insQ
49	*A. baumannii*	RO	1	32	≥64	64	≤0.12	8	≥256	≥256	≥64	1	ADC-75, OXA-69, OXA-23	ANT(2")-Ia, APH(3')-VIa	Omp33-36 ΔV44insT& A45insQ
50	*A. baumannii*	RO	2176	0.5	≤0.25	2	≤0.12	0.5	4	≤1	≤0.25	1	ADC-2, OXA-64		
52	*A. baumannii*	RO	1	32	≥64	64	≤0.12	8	≥256	128	32	1	ADC-75, OXA-69, OXA-23	ANT(2")-Ia, APH(3')-Ia, APH(3')-VIa	
53	*A. baumannii*	RO	1	32	≥64	64	≤0.12	16	≥256	≥256	32	1	IS*Aba1*-ADC-75, OXA-69, IS*Aba1*-OXA-23	ANT(2")-Ia, ANT(3")-IIa, APH(3')-Ia, APH(3')-VIa	Omp33-36 A45insQ Omp25 V234fs PBP3 E169fs
54	*A. baumannii*	RO	1	32	≥64	≥128	≤0.12	8	≥256	128	32	1	ADC-75, OXA-69, OXA-23	ANT(2")-Ia, APH(3')-Ia, APH(3')-VIa	
55	*A. baumannii*	RO	1	32	≥64	≥128	≤0.12	8	≥256	128	32	2	ADC-75, OXA-69, OXA-23	ANT(2")-Ia, APH(3')-Ia	
56	*A. baumannii*	RO	1	32	≥64	64	≤0.12	8	≥256	≥256	32	2	ADC-75, OXA-69, OXA-23	ANT(2")-Ia, APH(3')-Ia, APH(3')-VIa	OprD Q315fs
57	*A. baumannii*	AV	1470	≤0.25	0.5	1	0.25	0.5	8	≤1	≤0.25	1	ADC-58, OXA-106		
58	*A. baumannii*	AV	6	≤0.25	1	4	0.25	1	4	≤1	≤0.25	1	ADC-2, OXA-94		
61	*A. baumannii*	AV	1639	≤0.25	0.5	2	≤0.12	0.5	4	≤1	≤0.25	1	ADC-7, OXA-64		
62	*A. baumannii*	AV	265	≤0.25	≤0.25	1	≤0.12	1	2	≤1	≤0.25	1	ADC-2, OXA-91		
67	*A. baumannii*	AV	1	≤0.25	0.5	2	≤0.12	0.5	4	≤1	≤0.25	2	ADC-11, OXA-69		
79	*A. baumannii*	BA	106	≤0.25	≤0.25	8	≤0.12	0.5	4	≤1	≤0.25	1	ADC-11, OXA-78		
80	*A. baumannii*	BA	2182	≤0.25	1	2	≤0.12	2	4	≤1	0.5	1	ADC-6, OXA-208		

81	*A. baumannii*	BA	25	≤0.25	≤0.25	1	≤0.12	0.5	8	≤1	≤0.25	0.5	ADC-79, OXA-64		
83	*A. baumannii*	BA	25	16	≥64	32	≤0.12	8	16	4	32	2	ADC-5, OXA-64, OXA-23		
93	*A. baumannii*	BA	25	≤0.25	≤0.25	1	≤0.12	0.5	4	≤1	0.5	0.5	ADC-79, OXA-64		
95	*A. baumannii*	AM	582	≤0.25	0.5	1	≤0.12	0.5	2	≤1	≤0.25	0.5	ADC-5, OXA-317		
96	*A. baumannii*	AM	582	≤0.25	0.5	1	0.25	0.5	4	≤1	≤0.25	0.5	ADC-5, OXA-317		
100	*A. baumannii*	AR	145	≤0.25	0.5	1	≤0.12	0.5	≤1	≤1	≤0.25	0.5	ADC-58, OXA-343		
109	*A. baumannii*	RS	1	32	≥64	64	≤0.12	16	≥256	≥256	32	2	ADC-75, OXA-69, OXA-23	ANT(2'')-Ia, APH(3')-Ia, APH(3')-VIa	
110	*A. baumannii*	RS	1623	≤0.25	0.5	16	≤0.12	0.5	16	4	≤0.25	4	ADC-3, OXA-70		PmrC A46T
112	*A. baumannii*	RS	193	≤0.25	≤0.25	≤0.5	≤0.12	0.5	4	≤1	≤0.25	2	ADC-like, OXA-120		
124	*A. baumannii*	TP	2176	≤0.25	≤0.25	1	≤0.12	0.5	≤1	≤1	≤0.25	1	ADC-2, OXA-64		
127	*A. baumannii*	TP	2176	≤0.25	≤0.25	1	≤0.12	0.5	16	4	≤0.25	1	ADC-2, OXA-64		
128	*A. baumannii*	TP	2176	≤0.25	≤0.25	1	≤0.12	0.5	4	≤1	≤0.25	1	ADC-2, OXA-64		
130	*A. baumannii*	AC	32	≤0.25	1	4	≤0.12	1	4	≤1	≤0.25	0.5	ADC-79, OXA-100		
131	*A. baumannii*	JO	687	≤0.25	≤0.25	2	≤0.12	0.5	4	≤1	≤0.25	1	ADC-like, OXA-208		
132	*A. baumannii*	JO	1112	≤0.25	1	4	≤0.12	1	4	≤1	≤0.25	1	ADC-79, OXA-89		
133	*A. baumannii*	JO	2	16	8	32	0.25	32	64	128	32	2	ADC-30, OXA-66, OXA-58, TEM-12	AAC(3)-Ia, AAC(6')-Iaf, APH(3'')-Ib, APH(6)-Id	
134	*A. baumannii*	JO	2	16	8	32	≤0.12	32	32	64	8	2	ADC-30, OXA-66, OXA-58, TEM-12	AAC(6')-Iaf, ANT(3'')-IIa, APH(3'')-Ib, APH(6)-Id	
136	*A. baumannii*	AC	2183	≤0.25	0.5	4	≤0.12	0.5	8	≤1	≤0.25	1	ADC-like, OXA-66		
137	*A. baumannii*	PM	85	16	32	32	≤0.12	8	4	≤1	8	0.25	ADC-2, OXA-94, OXA-23		
138	*A. baumannii*	PM	745	32	16	32	0.5	16	64	8	≥64	2	ADC-30, OXA-66, OXA-58	APH(3'')-Ib, APH(6)-Id	
139	*A. baumannii*	PM	54	≤0.25	0.5	2	≤0.12	0.5	8	≤1	≤0.25	0.25	ADC-like, OXA-365		
140	*A. baumannii*	PM	85	16	≥64	32	0.25	8	16	≤1	16	≤0.12	ADC-2, OXA-94, OXA-23		

141	*A. baumannii*	PM	745	16	8	32	≤0.12	8	4	≤1	≥64	2	ADC-30, OXA-66, OXA-58	APH(3")-Ib, APH(6)-Id	
142	*A. baumannii*	PM	1	0.5	1	16	≤0.12	2	4	≤1	32	1	ADC-like, OXA-69		
143	*A. baumannii*	PM	1328	≤0.25	1	4	≤0.12	1	2	≤1	≤0.25	1	ADC-76, OXA-408		
144	*A. baumannii*	PM	745	16	16	32	≤0.12	4	128	16	≥64	1	ADC-30, OXA-66, OXA-58	APH(3")-Ib, APH(6)-Id	
148	*A. baumannii*	PM	745	32	8	32	0.25	16	≤1	≤1	≥64	2	ADC-30, OXA-66, OXA-58	APH(3")-Ib, APH(6)-Id	
149	*A. baumannii*	PM	1112	1	1	2	≤0.12	1	8	≤1	0.5	2	ADC-6, OXA-407		
150	*A. baumannii*	PM	2184	≤0.25	≤0.25	2	≤0.12	0.5	4	≤1	≤0.25	0.5	ADC-like, OXA-64		
152	*A. baumannii*	PM	132	≤0.25	≤0.25	1	≤0.12	0.5	≤1	≤1	≤0.25	0.5	ADC-2, OXA-120		
158	*A. baumannii*	SE	2034	≤0.25	≤0.25	2	≤0.12	0.5	≤1	≤1	≤0.25	0.5	ADC-2, OXA-217		
163	*A. baumannii*	SE	132	≤0.25	≤0.25	1	0.25	0.5	4	≤1	≤0.25	1	ADC-2, OXA-120		
167	*A. baumannii*	AS	1623	≤0.25	≤0.25	1	≤0.12	0.5	8	≤1	≤0.25	1	ADC-like, OXA-345		
168	*A. baumannii*	AS	1470	≤0.25	≤0.25	2	≤0.12	0.5	4	≤1	≤0.25	0.25	ADC-75, OXA-402		
169	*A. baumannii*	AS	2	32	≥64	32	0.25	16	≥256	≥256	≥64	2	ADC-30, OXA-66, OXA-23	AAC(6')-Ib7, ANT(3")-IIa, APH(3")-Ib, APH(6)-Id, ArmA	
170	*A. baumannii*	AS	2110	0.5	0.5	1	≤0.12	0.5	2	≤1	≤0.25	1	ADC-4, OXA-338		
171	*A. baumannii*	AS	2	16	32	32	≤0.12	8	≥256	≥256	≥64	≥128	ADC-30, OXA-66, OXA-23	AAC(6')-Ib7, ANT(3")-IIa, APH(3')-Ia, APH(3")-Ib, APH(6)-Id, ArmA	
172	*A. baumannii*	AS	2185	≤0.25	0.5	8	0.5	1	8	≤1	≤0.25	2	ADC-5, OXA-217		
173	*A. baumannii*	AS	2	32	32	≥128	2	32	8	≤1	≥64	4	IS*Aba1*-ADC-30, OXA-66, OXA-23	APH(3')-Ia, APH(3")-Ib, APH(6)-Id	IS*Aba1*-OpgE
174	*A. baumannii*	AS	2	32	≥64	≥128	≤0.12	8	≥256	≥256	≥64	0.5	ADC-30, OXA-66, OXA-23	AAC(6')-Ib7, ANT(3")-IIa, APH(3')-Ia, APH(3")-Ib, APH(6)-Id, ArmA	
179	*A. baumannii*	AX	2	32	≤64	≥128	≤0.12	16	≥256	≥256	≥64	0.5	ADC-30, OXA-66, OXA-23	AAC(6')-Ib7, ANT(3")-IIa, APH(3")-Ib, APH(6)-Id, ArmA	
180	*A. baumannii*	AX	2	16	16	32	≤0.12	2	2	≤1	≥64	4	ADC-30, OXA-66, OXA-24/40	APH(3")-Ib, APH(6)-Id	PmrB L94M

181	*A. baumannii*	AX	2	32	≥64	≥128	≤0.12	8	≥256	≥256	≥64	0.5	ADC-30, OXA-66, IS*Aba1*-OXA-23	AAC(6')-Ib7, ANT(3")-IIa, APH(3")-Ib, APH(6)-Id, ArmA		
182	*A. baumannii*	AX	2	32	≥64	≥128	≤0.12	4	≥256	≥256	≥64	0.5	ADC-30, OXA-66, OXA-23	AAC(6')-Ib7, ANT(3")-IIa, APH(3")-Ib, APH(6)-Id, ArmA		
183	*A. baumannii*	AX	2	32	≥64	≥128	≤0.12	8	≥256	≥256	≥64	1	ADC-30, OXA-66, IS*Aba1*-OXA-23	APH(3")-Ib, APH(6)-Id, ArmA		
184	*A. baumannii*	AX	2	32	≥64	≥128	1	≥64	≥256	≥256	≥64	0.5	ADC-30, OXA-66, IS*Aba1*-OXA-23	AAC(6')-Ib7, ANT(3")-IIa, APH(3")-Ib, APH(6)-Id, ArmA		
185	*A. baumannii*	AX	2	≤0.25	0.5	1	≤0.12	0.5	2	≤1	≤0.25	1	ADC-26, OXA-66			
186	*A. baumannii*	AX	2	≥64	≥64	64	≤0.12	8	≥256	≥256	≥64	1	ADC-30, OXA-66, OXA-23	AAC(6')-Ib7, ANT(3")-IIa, APH(3")-Ib, APH(6)-Id, ArmA		
187	*A. baumannii*	AX	105	0.5	≤0.25	1	≤0.12	0.5	4	≤1	≤0.25	1	ADC-2, OXA-430			
188	*A. baumannii*	AX	203	≤0.25	0.5	1	≤0.12	1	4	≤1	≤0.25	4	ADC-6, OXA-217, PDC-1			PmrA S119T
189	*A. baumannii*	AX	1405	0.5	0.5	2	≤0.12	1	2	≤1	≥64	1	ADC-3, OXA-120			
190	*A. baumannii*	AX	2186	≤0.25	≤0.25	1	≤0.12	0.5	4	≤1	≤0.25	1	ADC-6, OXA-413			
191	*A. baumannii*	AX	2	≥64	≥64	≥128	≤0.12	32	≥256	≥256	≥64	0.25	ADC-30, OXA-66, IS*Aba1*-OXA-23	AAC(6')-Ib7, ANT(3")-IIa, APH(3")-Ib, APH(6)-Id, ArmA		
192	*A. baumannii*	AX	2	≥64	≥64	32	≤0.12	8	≥256	≥256	≥64	0.5	ADC-30, OXA-66, IS*Aba1*-OXA-23	AAC(6')-Ib7, ANT(3")-IIa, APH(3")-Ib, APH(6)-Id, ArmA		
193	*A. baumannii*	AX	164	≤0.25	0.5	1	≤0.12	1	2	≤1	≤0.25	0.5	ADC-6, OXA-91			
194	*A. baumannii*	AX	2	≥64	≥64	32	≤0.12	16	≥256	≥256	≥64	2	ADC-30, OXA-66, OXA-23	AAC(6')-Ib7, ANT(3")-IIa, APH(3")-Ib, APH(6)-Id, ArmA		
195	*A. baumannii*	AX	1	0.5	0.5	2	≤0.12	0.5	2	≤1	≤0.25	2	ADC-11, OXA-69			
199	*A. baumannii*	AX	2	32	32	≥128	0.5	8	≥256	≥256	≥64	1	ADC-30, OXA-66, OXA-23	APH(3")-Ib, APH(6)-Id, ArmA		
200	*A. baumannii*	AX	1336	≤0.25	≤0.25	1	≤0.12	0.5	4	≤1	≤0.25	0.5	ADC-2, OXA-51			
201	*A. baumannii*	AX	2	32	≥64	≥128	≤0.12	8	≥256	≥256	≥64	0.25	ADC-30, OXA-66, OXA-23	AAC(6')-Ib7, ANT(3")-IIa, APH(3")-Ib, APH(6)-Id, ArmA	OprD Q33*	

202	*A. baumannii*	AX	1	≤0.25	≤0.25	2	≤0.12	0.5	≤1	≤1	0.5	2	ADC-11, OXA-69		
203	*A. baumannii*	AX	221	≤0.25	0.5	2	≤0.12	0.5	4	≤1	≤0.25	1	ADC-6, OXA-88		
204	*A. baumannii*	AX	2	16	32	16	≤0.12	8	2	4	≥64	1	ADC-30, OXA-66, OXA-23	AAC(6')-Ib7, APH(3'')-Ib, APH(6)-Id	CarO W57*
206	*A. baumannii*	AC	2187	≤0.25	≤0.25	1	≤0.12	0.5	4	≤1	≤0.25	2	ADC-58, OXA-69		

IMI, imipenem; MEM, meropenem; FEP, cefepime; SUL, sulbactam; AMK, amikacin; TOB, tobramycin; CIP, ciprofloxacin; COL, colistin; FDC, cefiderocol.

OpgE; lipid A phosphoethanolamine transferase

Shaded cells indicate MIC values above the resistance breakpoint according to CLSI guidelines.

Site's abbreviations: AC (A Coruna University Hospital), VM (University Hospital Virgen Macarena), VN (University Hospital Virgen de las Nieves), UC (Canary Islands University Hospital), VA (University Clinical Hospital of Valencia), RO (University Hospital Virgen del Rocío), AV (University Hospital Arnau de Vilanova), BA (Basurto University Hospital), AM (Hospital Arquitecto Marcide), AR (Araba University Hospital), RS (University Hospital Reina Sofía), TP (University Hospital Germans Trias i Pujol), JO (University Hospital Joan XXIII), PM (University Hospital Puerta del Mar), SE (University Hospital Son Espases), AS (Asturias Central University Hospital), AX (University Clinical Hospital Virgen de la Arrixaca).

[a]**Mutation's abbreviations**: premature stop codon (*); deletion (Δ); frameshift (fs); insertion (ins).

Table S4. Species identification, isolation site, MIC values and putative resistance mechanisms for the set of *A.* non-*baumannii* isolates, Spain, 2020 (n=81).

ID	Species	Site	MIC (mg/L)									Putative resistance mechanisms		
			IMI	MEM	FEP	FDC	SUL	AMK	TOB	CIP	COL	beta-lactamases	Other	PmrCAB and related systems
2	*A. dispersus*	AC	≤0.25	0.5	2	≤0.12	0.5	8	4	≤0.25	16	OXA-291	AAC(6')-Ix, aadA27	PmrB E103K, T180A, A191V<br>PmrC S75A
3	*A. ursingii*	AC	≤0.25	0.5	2	≤0.12	≤0.25	4	2	≤0.25	0.5			
27	*A. soli*	AC	≤0.25	≤0.25	≤0.5	≤0.12	0.5	≤1	≤1	≤0.25	0.25			
43	*A. nosocomialis*	AC	≤0.25	0.5	4	0.5	1	4	≤1	1	0.5	ADC-131		
59	*A. pittii*	AV	≤0.25	0.5	2	1	0.5	2	≤1	≤0.25	1	ADC-70, OXA-500		
60	*A. dijkshoorniae*	AV	≤0.25	0.5	2	1	0.5	2	≤1	≤0.25	0.5	ADC-70, OXA-500		
63	*A. dijkshoorniae*	AV	0.5	1	2	0.25	0.5	4	≤1	≤0.25	0.5	ADC-16, OXA-270		
64	*A. ursingii*	AV	≤0.25	0.5	4	1	1	≤1	≤1	≤0.25	1		APH(3'')-Ib, APH(3')-Ia, APH(6)-Id	
65	*A. dijkshoorniae*	AV	≤0.25	1	2	0.25	0.5	4	≤1	≤0.25	2	ADC-245, OXA-506		
66	*A. nosocomialis*	AV	32	≥64	8	0.5	2	8	4	≤0.25	0.5	ADC-131, OXA-24	AAC(3)-IIe, APH(3'')-Ib, APH(6)-Id, aadT	
68	*A. pittii*	AV	≤0.25	1	8	2	0.5	4	≤1	0.5	0.5	ADC-18, OXA-821		
69	*A. guillouiae*	AV	0.5	1	4	0.25	0.5	2	≤1	≤0.25	1		APH(3')-VIb	
70	*A. bereziniae*	AV	≤0.25	0.5	1	≤0.12	0.5	≤1	≤1	≤0.25	2	OXA-301		
71	*A. ursingii*	AV	≤0.25	≤0.25	4	≤0.12	0.5	≤1	≤1	≤0.25	0.25		ANT(3'')-IIc	
72	*A. haemolyticus*	AV	≤0.25	0.5	2	0.5	0.5	16	64	≤0.25	2	OXA-674	AAC(6')-It, ANT(2'')-Ia, ANT(3'')-IIc	

73	*A. pittii*	AV	≤0.25	0.5	2	≤0.12	0.5	2	≤1	≤0.25	1	ADC-18, OXA-500		
74	*A. guillouiae*	AV	≤0.25	0.5	2	0.25	≤0.25	2	≤1	≤0.25	1	OXA-669	APH(3')-VIa	
75	*A. pittii*	AV	≤0.25	0.5	4	≤0.12	0.5	4	≤1	≤0.25	1	ADC-252, OXA-943		
76	*A. junii*	AV	≤0.25	≤0.25	2	≤0.12	0.5	≤1	≤1	≤0.25	2		AAC(6')-Ir,	
77	*A. junii*	AV	32	≥64	2	1	1	2	2	1	2	OXA-24	AAC(3)-IIe, APH(3'')-Ib, APH(6)-Id, aadT	
78	*A. pittii*	BA	0.5	≤0.25	2	≤0.12	0.5	4	≤1	≤0.25	0.5	ADC-245, OXA-506		
82	*A. pittii*	BA	≤0.25	≤0.25	1	0.25	0.5	4	≤1	≤0.25	0.5	ADC-70, OXA-417		
84	*A.bereziniae*	BA	≤0.25	0.5	1	≤0.12	0.5	≤1	≤1	≤0.25	2	OXA-355		
85	*Acinetobacter spp.*[a]	BA	≤0.25	1	4	1	1	4	≤1	≤0.25	1	ADC-134, OXA-323		
86	*A. pitii*	BA	≤0.25	0.5	2	≤0.12	0.5	4	≤1	≤0.25	0.5	ADC-245, OXA-506		
87	*A. ursingii*	BA	≤0.25	≤0.25	≤0.5	≤0.12	≤0.25	≤1	≤1	≤0.25	0.25			
88	*Acinetobacter spp.*[b]	BA	≤0.25	≤0.25	≤0.5	0.25	1	2	≤1	≤0.25	≤0.12	ADC-202, OXA-421		
89	*Acinetobacter spp.*[b]	BA	≤0.25	≤0.25	≤0.5	1	1	2	≤1	≤0.25	≤0.12	ADC-202, OXA-421		
90	*A. pittii*	BA	≤0.25	≤0.25	≤0.5	≤0.12	0.5	2	≤1	≤0.25	0.25	ADC-245, OXA-506		
91	*A. pittii*	BA	≤0.25	≤0.25	2	≤0.12	1	4	≤1	≤0.25	0.5	ADC-245, OXA-506		
92	*A. haemolyticus*	BA	≤0.25	≤0.25	2	≤0.12	0.5	16	8	2	1	OXA-264	AAC(6')-Ig	
94	*A. calcoaceticus*	BA	≤0.25	0.5	2	0.5	0.5	2	≤1	≤0.25	0.5	ADC-194, OXA-844		
97	*A. pittii*	AM	≤0.25	0.5	4	2	0.5	4	≤1	≤0.25	2	ADC-18, OXA-500		

98	*A. nosocomialis*	AM	≤0.25	0.5	1	0.25	0.5	4	≤1	≤0.25	1	ADC-130		
99	*Acinetobacter* spp.[c]	AM	≤0.25	0.5	2	1	0.5	4	2	≤0.25	4	OXA-303	AAC(6')-Ix, ANT(3'')-IIc	
101	*A. johnsonii*	AR	≤0.25	≤0.25	2	≤0.12	≤0.25	≤1	≤1	≤0.25	0.25	OXA-334		
102	*Acinetobacter* spp.[c]	AR	≤0.25	0.5	2	≤0.12	0.5	2	≤1	≤0.25	4	OXA-303	AAC(6')-Ix, ANT(3'')-IIc	
103	*A. ursingii*	AR	≤0.25	≤0.25	4	≤0.12	0.5	≤1	≤1	≤0.25	1			
104	*A.bereziniae*	AR	≤0.25	0.5	1	≤0.12	0.5	≤1	≤1	≤0.25	2	OXA-356		
105	*A. pittii*	AR	≤0.25	≤0.25	2	≤0.12	0.5	4	≤1	≤0.25	0.5	ADC-150, OXA-421		
106	*A. pittii*	AR	≤0.25	≤0.25	1	≤0.12	0.5	4	≤1	≤0.25	1	ADC-150, OXA-777		
107	*A. pittii*	AR	≤0.25	0.5	1	0.25	0.5	4	≤1	≤0.25	0.5	ADC-70, OXA-500		
108	*A. calcoaceticus*	RS	≤0.25	≤0.25	2	0.25	0.5	≤1	≤1	≤0.25	0.5			
111	*A. pittii*	RS	≤0.25	≤0.25	1	≤0.12	0.5	4	≤1	≤0.25	1			
113	*A. pittii*	RS	≤0.25	≤0.25	1	≤0.12	0.5	4	≤1	≤0.25	0.5			
114	*A. bereziniae*	RS	≤0.25	0.5	≤0.5	≤0.12	0.5	≤1	≤1	1	2	OXA-355		
115	*A. bereziniae*	RS	≤0.25	0.5	1	≤0.12	0.5	≤1	≤1	≤0.25	2	OXA-301		
116	*A. bereziniae*	RS	≤0.25	≤0.25	1	≤0.12	0.5	≤1	≤1	≤0.25	4	OXA-355		PmrC I103V, S117P, VS133FG, V233I, V272A, A301V, H285Q, A360S
117	*A. bereziniae*	RS	≤0.25	0.5	8	≤0.12	0.5	8	2	≤0.25	8			PmrC Y78N, V233I, VD272AN, A301V, Y323I
118	*A. pittii*	RS	≤0.25	0.5	2	≤0.12	0.5	4	≤1	≤0.25	0.5			
119	*A. ursingii*	RS	≤0.25	≤0.25	8	≤0.12	1	≤1	≤1	≤0.25	0.5			
120	*A. ursingii*	RS	≤0.25	0.5	16	≤0.12	0.5	2	≤1	≤0.25	0.25			
121	*A. pitii*	RS	≤0.25	0.5	2	≤0.12	0.5	2	≤1	≤0.25	1	ADC-201, OXA-843		

122	*A. beijerinckii*	RS	≤0.25	≤0.25	2	0.5	0.5	2	≤1	≤0.25	8			PmrC V46I, Q124H, V163I, S191G, M218V, V222E, MV226YA, V485I, PmrB N169S, Q196K, Q200R
123	*A. ursingii*	AC	≤0.25	≤0.25	1	≤0.12	0.5	4	≤1	≤0.25	1			
125	*A. pitii*	TP	≤0.25	≤0.25	2	≤0.12	0.5	4	≤1	≤0.25	1	ADC-150, OXA-421		
126	*A. ursingii*	TP	≤0.25	0.5	16	≤0.12	2	2	≤1	≤0.25	0.5		ANT(3")-IIc	
129	*A. bereziniae*	TP	≤0.25	0.5	1	≤0.12	0.5	≤1	≤1	≤0.25	2	OXA-356		
135	*A. pittii*	AC	≤0.25	≤0.25	1	≤0.12	0.5	4	≤1	≤0.25	1			
145	*A. pittii*	PM	≤0.25	1	2	≤0.12	0.5	4	≤1	≤0.25	1	ADC-150, OXA-421		
146	*A. lwoffii*	PM	≤0.25	≤0.25	≤0.5	≤0.12	≤0.25	≤1	≤1	≤0.25	≤0.12	OXA-134		
147	*A. ursingii*	PM	≤0.25	≤0.25	2	≤0.12	≤0.25	2	≤1	≤0.25	≤0.12		ANT(3")-IIc	
151	*A. pittii*	PM	≤0.25	0.5	4	≤0.12	0.5	≤1	≤1	≤0.25	0.5	ADC-18, OXA-821		
154	*A. pittii*	PM	0.5	≤0.25	1	≤0.12	0.5	≤1	2	≥64	0.5	ADC-18, OXA-500		
155	*A. ursingii*	AC	≤0.25	1	4	≤0.12	1	2	≤1	≤0.25	0.5			
156	*A. bereziniae*	SE	≤0.25	0.5	≤0.5	≤0.12	≤0.25	≤1	≤1	≤0.25	1	OXA-355		
157	*A. pittii*	SE	≤0.25	1	2	≤0.12	0.5	≤1	≤1	≤0.25	1	ADC-21, OXA-506		
159	*A. lwoffii*	SE	≤0.25	≤0.25	≤0.5	0.5	≤0.25	≤1	≤1	≤0.25	≤0.12	OXA-283		
160	*A. pittii*	SE	≤0.25	≤0.25	2	≤0.12	0.5	2	≤1	≤0.25	0.5	ADC-245, OXA-506		
161	*Acinetobacter* spp.[a]	SE	≤0.25	0.5	2	2	2	4	≤1	≤0.25	2	ADC-193		
162	*A. pittii*	SE	≤0.25	0.5	8	≤0.12	≤0.25	32	8	0.5	1	ADC-245 OXA-506		

164	*A. pittii*	SE	≤0.25	0.5	2	0.25	0.5	8	≤1	≤0.25	1	ADC-150, OXA-421		
165	*A. ursingii*	SE	≤0.25	≤0.25	4	0.25	1	2	≤1	≤0.25	0.5			
166	*A. ursingii*	SE	≤0.25	≤0.25	4	≤0.12	1	2	≤1	≤0.25	0.5			
175	*A. radioresistens*	AC	≤0.25	≤0.25	≤0.5	≤0.12	1	2	≤1	≤0.25	0.5	OXA-23		
176	*A. colistiniresistens*	HN	≤0.25	≤0.25	1	≤0.12	0.5	4	2	≥64	32	OXA-307	AAC(6')-Ij	
177	*A. haemolyticus*	AM	≤0.25	≤0.25	2	0.25	0.5	8	4	≤0.25	4	OXA-215	AAC(6')-Ig	
178	*A. pittii*	AC	≤0.25	≤0.25	4	≤0.12	0.5	8	≤1	≤0.25	0.5	ADC-18, OXA-821		
197	*A. pittii*	AX	≤0.25	0.5	1	≤0.12	0.5	4	≤1	≤0.25	1	ADC-18, OXA-500		
198	*A. calcoaceticus*	AX	≤0.25	≤0.25	2	0.25	0.5	2	≤1	≤0.25	0.5	ADC-194, OXA-822		
205	*A. calcoaceticus*	AC	≤0.25	0.5	2	1	2	≤1	≤1	≤0.25	2			

IMI, imipenem; MEM, meropenem; FEP, cefepime; SUL, sulbactam; AMK, amikacin; TOB, tobramycin; CIP, ciprofloxacin; COL, colistin; FDC, cefiderocol.

Shaded cells indicate MIC values above the resistance breakpoint according to CLSI guidelines.

Site's abbreviations: AC (A Coruna University Hospital), AV (University Hospital Arnau de Vilanova), BA (Basurto University Hospital), AM (Hospital Arquitecto Marcide), AR (Araba University Hospital), RS (University Hospital Reina Sofía), TP (University Hospital Germans Trias i Pujol), PM (University Hospital Puerta del Mar), SE (University Hospital Son Espases), HN (Naval Hospital), AX (University Clinical Hospital Virgen de la Arrixaca).

[a,b,c] *In* silico rMLST did not provide a 100 % identity with a specific *Acinetobacter* species. The closest species identity is as follows:

Acinetobacter spp. [a]: *A. calcoaceticus (79%), A. oleivorans (14%).*

Acinetobacter spp. [b]: *A. geminorum* (77%), *A. pittii* (22%).

Acinetobacter spp.[c]: *A. dispersus* (45%), *A. colistiniresistens* (18%), *A. proteolyticus* (18%), *A. gyllenbergii* (9%), *A. modestus* (9%).

Table S5. MIC distributions and resistance rates to the antibiotics tested, according to EUCAST v12.0 breakpoints and relative to the set of *A. baumannii* isolates, Spain, 2020 (n= 118).

Class of antibiotic	Per cent of isolates at MIC (mg/L)												% R (EUCAST v12.0)
	≤0.12	0.25	0.5	1	2	4	8	16	32	64	128	≥256	
**Beta-lactams**													
IMI		36.4[a]	41.5	42.4	42.4	43.2	<u>51.7</u>	65.2	**96.6**	100[b]			56.8
MEM		19.5[a]	35.6	42.4	42.4	42.4	46.6	<u>53.4</u>	66.9	**100[b]**			53.4
FEP			1.7[a]	19.5	34.7	39.8	41.5	<u>50.0</u>	76.3	88.1	**100[b]**		NA
FDC	<u>75.4</u>	**91.5**	96.6	98.3	100								NA
SUL[c]		0.0[a]	31.4	39.8	47.6	<u>56.8</u>	83.9	**95.8**	99.1	100[b]			NA
**Aminoglycosides**													
AMK				5.9[a]	17.8	<u>53.4</u>	61.9	67.8	69.5	71.2	72.0	**100**	38.1
TOB				<u>56.8[a]</u>	57.6	61.9	62.7	66.9	67.8	70.3	76.3	**100**	38.1
**Fluoroquinolones**													
CIP			36.4[a]	39.8	39.8	40.7	40.7	42.4	43.2	<u>52.5</u>	**100[b]**		60.2
**Polymixins**													
COL	0.8	5.9	23.7	<u>58.5</u>	86.4	**94.1**	99.1	99.1	99.1	99.1	100[b]		13.6

AMK: amikacin; CIP: ciprofloxacin; COL: colistin; FDC: cefiderocol; FEP: cefepime; IMI: imipenem; MEM: meropenem; MIC: minimum inhibitory concentration; NA: not available; R: resistance; SUL: sulbactam; TOB: tobramycin.

[a] Actual MIC value is equal or inferior to that stated.

[b] Actual MIC value is equal or higher to that stated.

[c] We considered sulbactam a beta-lactam in this context, due to its intrinsic antibacterial activity against *Acinetobacter.*

MIC_{50} underlined, MIC_{90} in bold.

Figure S1. Phylogenetic tree showcasing the predominant species of *A.* non-*baumannii*, *A. pittii* (A) and *A. ursingii* (B), Spain, 2020.

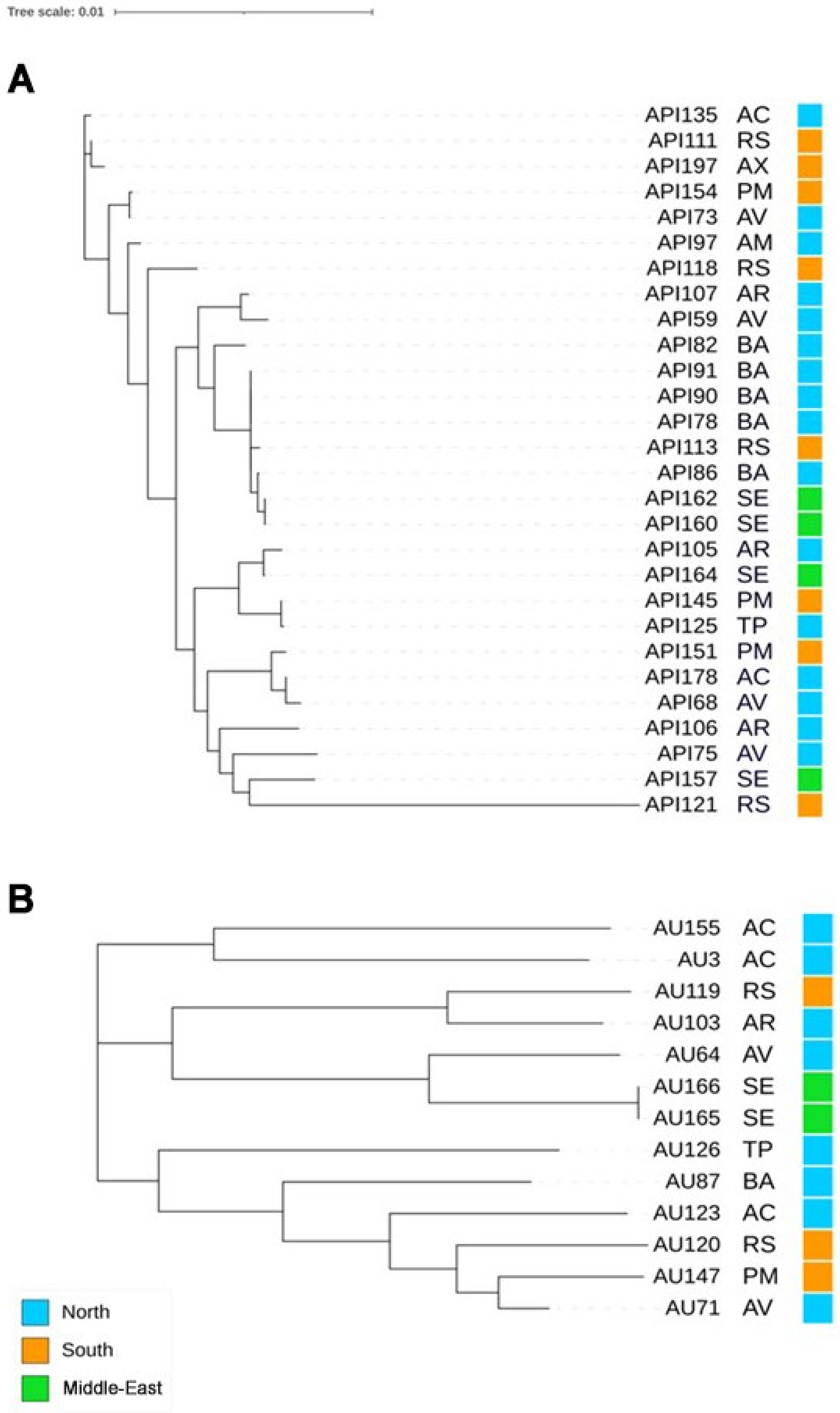

Materials and Methods

Molecular typing, phylogenetic analysis and antimicrobial resistance analysis.

The assemblies were submitted to the PubMLST ribosomal Multilocus Sequence Typing (rMLST) database (https://pubmlst.org/) for species of *Acinetobacter spp.* and sequence type (ST) determination, following the Pasteur scheme [1]. Additionally, CARD v3.2.5 and Resfinder v4.1 databases were used to assess the presence or absence of antimicrobial resistance (AMR) genetic determinants. The polished reads of *A. baumannii* were mapped against the reference genome *A. baumannii* ATCC 17978 using Snippy v4.6.0 (https://github.com/tseemann/snippy) and default parameters.

In order to assess mutations affecting antibiotic activity, the following genes were analyzed: *oprD, omp33-36, carO, ompC, omp25*, *pbpA* and *ftsI* (carbapenem resistance)*; gyrA* and *parC* (quinolone resistance); *pmrCAB* and the *lpx* operon (colistin resistance). Finally, IS*Aba* sequences upstream of *eptA*, *pmrCAB*, bla_{ADCs} and bla_{OXAs}, were also examined.

To assess genomic changes implicated in antimicrobial resistance in *A.* non-*baumannii* isolates, a comparison was performed against genomes of susceptible strains that were obtained from one of the following three methods: i) a susceptible strain from our collection, ii) ATCC strains with public genomes, or iii) a reference genome for that species in the NCBI taxonomy. Genes homologous to those cited above for *A. baumannii* were evaluated in the rest of the *Acinetobacter* spp.

Allele calling for *A. baumannii* was carried out using chewBBACCA [2], employing the cgMLST schema provided within the same software. After obtaining a cgMLST for the 118 *A. baumannii isolates*, a distance matrix was constructed based on the cgMLST allele calls to clarify the genomic relatedness among the isolates. *A. baumannii* isolates with a cgMLST (Allele) distance of ≤3 were categorized as closely related genomes [3]. Hence, the threshold of ≤3 cgMLST (Allele) was established to assess potential clonal dissemination among isolates. The phylogenetic dendrogram was created using the Neighbor-joining model with Ape package v.5.7-1 [4] available in the CRAN repository. Lastly, the tree was visualized with Itol software [5].

Allele calling for *A.* non-*baumannii* species was not possible because the cgMLST scheme has not yet been developed. Therefore, analyses of the phylogenetic trees of these species were performed. Genomes of *A.* non-*baumannii* were annotated using Bakta and then the core genome was identified using the pangenome clustering tool Panaroo [6]. Finally, the resulting phylogenetic tree was constructed with with RAxML using GTR+G model (discrete GAMMA model of rate heterogeneity with 4 categories) [7] and visualized through Itols software.

References

1. Diancourt L, Passet V, Nemec A, Dijkshoorn L, Brisse S. The population structure of *Acinetobacter baumannii:* Expanding multiresistant clones from an ancestral susceptible genetic pool. PLoS One. 2010;5(4):e10034.

2. Silva M, Machado MP, Silva DN, Rossi M, Moran-Gilad J, Santos S, et al. chewBBACA: A complete suite for gene-by-gene schema creation and strain identification. Microb genomics. 2018;4(3):1–7.

3. Schürch AC, Arredondo-Alonso S, Willems RJL, Goering R V. Whole genome sequencing options for bacterial strain typing and epidemiologic analysis based on single nucleotide polymorphism versus gene-by-gene–based approaches. Clin Microbiol Infect. 2018;24(4):350–4.

4. Paradis E, Schliep K. ape 5.0: an environment for modern phylogenetics and evolutionary analyses in R. Bioinformatics 35: 526-528. Bioinformatics. 2018;35(July):526–8.

5. Letunic I, Bork P. Interactive tree of life (iTOL) v5: An online tool for phylogenetic tree display and annotation. Nucleic Acids Res. 2021;49(W1):W293–6.

6. Tonkin-Hill G, MacAlasdair N, Ruis C, Weimann A, Horesh G, Lees JA, et al. Producing polished prokaryotic pangenomes with the Panaroo pipeline. Genome Biol. 2020;21(1):1–21.

7. Kozlov AM, Darriba D, Flouri T, Morel B, Stamatakis A. RAxML-NG: A fast, scalable and user-friendly tool for maximum likelihood phylogenetic inference. Bioinformatics. 2019;35(21):4453–5.
